# Immersive virtual reality with synchronous neurostimulation for upper-limb recovery after stroke: a randomized feasibility trial

**DOI:** 10.1038/s41591-026-04486-4

**Published:** 2026-06-26

**Authors:** Giuseppe Valerio Aurucci, Olivera Djordjevic, Andrea Cimolato, Natalija Secerovic, Tijana Dimkic Tomic, Maria Dolores Ardura Carnicero, Haotian Yao, Ljubica Konstantinovic, Stanisa Raspopovic

**Affiliations:** 1 Neuroengineering Laboratory, Department of Health Sciences and Technology, ETH Zürich, Zürich, Switzerland; 2 Clinic for Rehabilitation ‘Dr. Miroslav Zotović’, Belgrade, Serbia; 3 Faculty of Medicine, University of Belgrade, Belgrade, Serbia; 4 Neuroengineering Lab, Center for Medical Physics and Biomedical Engineering, Medical University of Vienna, Vienna, Austria; 5 Institute Mihajlo Pupin, Belgrade, Serbia

**Keywords:** Stroke, Brain injuries, Translational research, Rehabilitation

## Abstract

Stroke affects 15 million people annually and leaves 5 million permanently disabled. In the chronic phase (>3 months after stroke), patients often experience persistent sensorimotor deficits and altered body representation, yet rehabilitation delivery remains partial and inconsistent, highlighting an unmet clinical need. Immersive technologies and noninvasive neurostimulation offer potential for scalable, intensive rehabilitation, but clinical evidence supporting multimodal approaches and objective outcome assessments remains limited. In this study, we evaluate the feasibility, clinical efficacy and assessment capabilities of a multimodal platform (MultiSensy) integrating virtual reality with synchronous transcutaneous sensory neurostimulation. Thirty-four patients with chronic stroke were enrolled in a combined pilot study (*n* = 9) and randomized, 33-day-long feasibility study (*n* = 25), where MultiSensy intervention was evaluated against conventional rehabilitation. Primary endpoints included motor function assessed by Fugl-Meyer Assessment Upper Extremity (FMA-UE) and Action Research Arm Test (ARAT) and self-body representation assessed by the Body Landmark Test. Secondary outcomes included sensory and functional independence evaluation. Continuous kinematic data were collected to derive objective performance markers. Compared to conventional rehabilitation, MultiSensy resulted in greater motor improvement, reflected by higher FMA-UE (13.17 ± 1.30 versus 7.54 ± 1.48; *P* = 0.01) and ARAT (8.25 ± 1.96 versus 2.44 ± 1.08; *P* = 0.029) scores. MultiSensy further improved body self-representation and hand tactile acuity. The platform enabled continuous performance monitoring and extraction of objective kinematic markers that tracked rehabilitation progress. These findings pave the way for larger trials and highlight the potential treatment of multiple patients with fewer physiotherapist visits needed or even home-based sensorimotor rehabilitation. ClinicalTrials.gov identifier: NCT06400823.

## Abstract (web summary)

A multimodal platform combining immersive virtual reality with a synchronous sensory neurostimulation protocol outperformed conventional rehabilitation in individuals with chronic stroke, driving upper-limb motor gains, restored body representation and improved tactile acuity while enabling objective kinematic tracking of recovery.

## Main

Stroke affects 15 million individuals annually, making it the second-leading cause of death and the third-leading cause of disability worldwide^1,2^. At the time of hospital admission, stroke survivors face immediate and often severe sensorimotor impairments that affect walking, coordination and speech, resulting in an abrupt loss of autonomy and functional independence^3^. Despite early rehabilitation efforts to restore cognitive and motor functions, many patients are discharged with persistent deficits that limit their ability to participate in social and professional life^4^. Notably, clinical evidence demonstrates that recovery extends beyond the acute phase, as patients can significantly improve their clinical status even several months after stroke^5,6^. At the chronic phase, stroke survivors still experience persistent hemiparetic sensorimotor deficits, such as spasticity, muscle weakness, loss of coordination^7^ and impaired tactile perception on the affected side^8^. Moreover, various evidence showed how stroke chronically induces maladaptive plastic changes^9–11^, critically resulting in chronic alterations in movement planning and execution^12^ and distorted body representation^13–18^ (that is, brain-encoded position and angles of the body^19^). Namely, for upper-limb impairment, for instance, it was shown^15^ that patients with chronic stroke misperceive their impaired arm dimensions and have reduced peripersonal space (PPS) (that is, the space in which multisensory stimuli integration is facilitated^20,21^). However, despite the multidimensionality of stroke deficits across sensory and body representation domains, rehabilitation remains predominantly motor focused^22^.

In clinical practice, the most common rehabilitation strategy is task-specific training, involving repetitive, goal-directed movements to promote functional recovery and independence. This includes occupational therapy, which targets activities of daily living (ADLs)^23^, and constraint-induced movement therapy (CIMT)^24^, which promotes the use of the impaired limb by restricting the unaffected one^25^. However, task-specific training alone may be insufficient to support full recovery. It could be strengthened by priming techniques^26^, such as transcranial direct current stimulation or transcutaneous electrical nerve stimulation (TENS), which enhance neural excitability and prepare the brain for relearning, and by augmenting strategies that directly target motor impairments using, for instance, robotic devices. On the other side, functional electrical stimulation (FES) has long been applied in post-stroke upper-limb rehabilitation to restore voluntary movement and prevent disuse-related muscle atrophy^27^. Several studies have demonstrated clinically meaningful improvements in FMA-UE^28^ and ARAT^29^ scores after hybrid FES–robotic^30,31^. However, although effective for motor reactivation, these systems often lack concurrent sensory feedback, limiting their ability to fully engage sensorimotor integration mechanisms crucial for functional recovery.

Despite their potential, integrated multimodal stroke rehabilitation applications remain rare. Moreover, in real-world practice, rehabilitation type and, more importantly, dosage become highly inconsistent after hospital discharge, leading to tremendous post-acute rehabilitation intensity variability across patients^23,32^.

To address these challenges, health agencies advocate for innovative multisensory approaches that integrate diverse stroke rehabilitation strategies while delivering high-dosage therapy with minimal supervision, hence reducing the burden on healthcare systems. In this view, virtual reality (VR) can deliver personalized rehabilitation exercises with minimal supervision by medical personnel, accelerating patients’ functional recovery^33^. Moreover, VR is known to enhance user motivation and engagement through immersive, goal-directed tasks^34^. This engagement not only improves adherence but also neurophysiologically activates reinforcement-related circuits^35,36^, including the ventral tegmental area projecting to the motor cortex^37–40^, supporting neuroplastic processes critical for motor recovery. Although evidence regarding the efficacy of VR over conventional rehabilitation remains inconclusive^41^ due to the diversity of VR applications (for example, type, dosage and timing), growing research highlights the benefits of purpose-designed immersive VR^42^. Integrating tactile cues into immersive visuomotor training provides additional somatosensory information that reinforces sensorimotor coupling^34^ and offers real-time, task-contingent feedback, an essential component of effective motor relearning in stroke rehabilitation^43^. In this context, TENS emerges as a noninvasive and cost-effective method for delivering sensory feedback while retraining afferent fibers of the affected limb^44,45^. TENS has demonstrated efficacy in supporting both sensory and motor recovery, leveraging the critical role of somatosensory input for motor skill acquisition^22,46^. More crucially, the integration of tactile feedback within immersive VR environments deepens user immersion through synchronous multisensory contingencies^47–49^, a key driver of the ‘body ownership illusion’^50^ (the sense of inhabiting an alternate body^51,52^). In principle, this may directly target somatosensory-encoded body representation, hence addressing sensorimotor conflicts and promoting functional recovery^12,53^. Although recent studies have combined virtual or augmented reality with various forms of noninvasive brain or FES stimulation^54–57^, none has yet implemented fully immersive VR integrated with synchronized targeted sensory neurostimulation over multi-week rehabilitation training. Consequently, the potential of immersive VR coupled with peripheral somatosensory stimulation to reinforce body ownership illusions and support motor recovery remains largely unexplored.

Alongside their rehabilitation potential, immersive multimodal systems offer the unique opportunity of continuously tracking participants’ kinematics and body perception status during training (via built-in cameras)^58^. These objective indicators can be integrated with clinically validated measures, such as the FMA-UE^28^ and the ARAT^29^, which evaluate sensorimotor impairment and functional performance in patients with stroke. Combining kinematic and body perception metrics with these tools may offer clinicians a precise and comprehensive profile of a patient’s recovery^59^, enabling data-driven personalization of rehabilitation strategies.

To these aims, we constructed MultiSensy: a multimodal immersive platform that integrates VR scenarios with congruent targeted sensory neurostimulation (electro-tactile feedback perceived at the site of virtual contact and precisely time synchronous with the interactions with virtual objects) to support sensorimotor rehabilitation and assessment in chronic stroke survivors. The platform was tested on 34 stroke survivors in two stages: a pilot study to assess usability and short-term effects, followed by a randomized feasibility study serving as a proof-of-concept intervention to determine MultiSensy clinical efficacy and impact on motor, body representation and sensory impairments.

## Results

### Study design and patient demographics

**Fig. 1:**
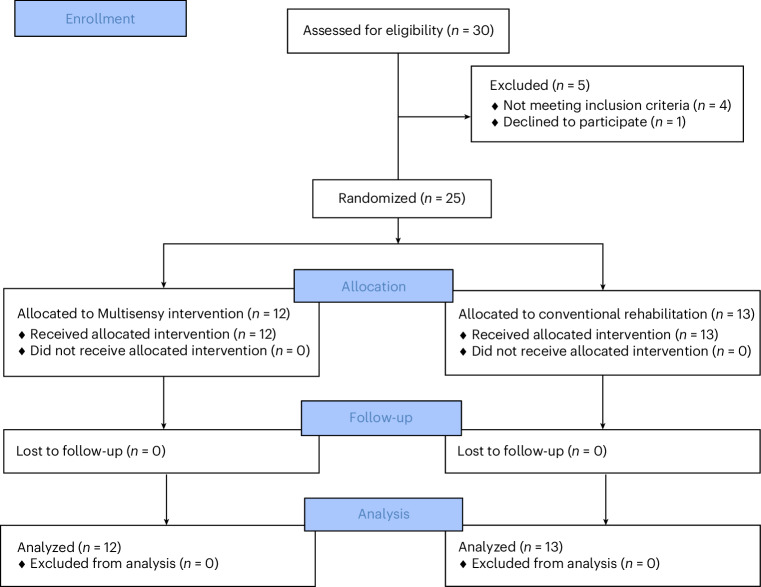
CONSORT diagram. CONSORT flow diagram of participant enrollment, allocation, follow-up and analysis in the randomized feasibility study.

A total of 34 stroke survivors were included in two stages: a pilot study (*n* = 9) to assess usability and the platform’s short-term effects, followed by a randomized feasibility study (*n* = 25) to examine its clinical efficacy and rehabilitative impact. Nine participants (one female, eight males; mean age 59 ± 8 years; inclusion and exclusion criteria in Extended Data Table 1) were enrolled in the pilot study. The demographic and clinical characteristics of participants in the pilot phase are summarized in Extended Data Table 2. Twenty-five chronic stroke survivors (nine females, 16 males; mean age 59 ± 10 years) were recruited for the randomized feasibility study (CONSORT diagram in Fig. 1; inclusion/exclusion criteria in Extended Data Table 1). Detailed sample sizes per outcome are provided in Supplementary Table 1.

**Extended Data Fig. 1:**
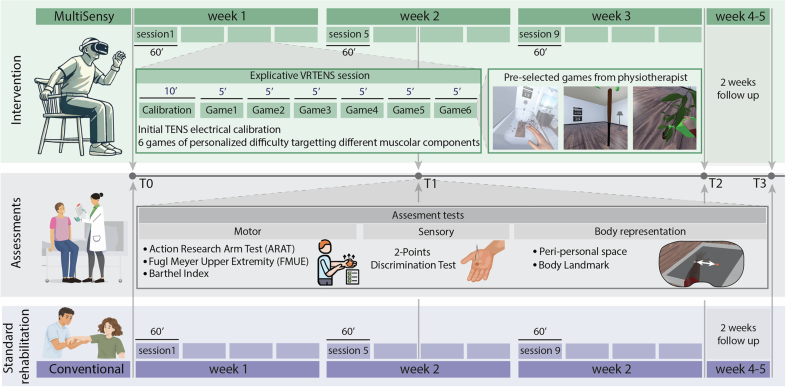
Randomized feasibility study protocol. Participants were randomized into two groups: MultiSensy and Conventional Rehabilitation. Both groups underwent 12 rehabilitation sessions over three weeks, matched in dose and type of movements performed. In the MultiSensy group, each session began with an electrical stimulation calibration, followed by 6–8 personalized rehabilitation games, each lasting 4 min. Assessments were conducted at T0 (Day 1, baseline, pre-intervention), T1 (Day 10, after six sessions), T2 (Day 19, post-intervention), and T3 (Day 33, follow-up, two weeks post-intervention). The assessment battery included motor-functional tests (FMUE, ARAT, Barthel Index), sensory evaluation (2PD), and body representation tests (Body Landmark and Peri-Personal Space).

The recruitment of the randomized feasibility study occurred between April 2024 and April 2025. Participants were randomized into two groups: MultiSensy (*n* = 12) or conventional rehabilitation (*n* = 13). Both groups underwent 3 weeks of dose-matched upper-limb rehabilitation, corresponding to 12 sessions (four per week) of active intervention. The MultiSensy group used the immersive neurostimulation platform, completing 6–8 rehabilitation games per session, each lasting at least 4 minutes (Extended Data Fig. 1). The conventional rehabilitation group participated in therapist-supervised sessions, matched in duration and movement types to the MultiSensy sessions (Extended Data Fig. 1). Assessments were conducted at baseline (day 1, T0), midway through the intervention (day 10, T1), at the end (day 19, T2) and at 2-week post-intervention follow-up (day 33, T3).

The demographic (sex and age), clinical (months since stroke, impaired arm, FMA-UE and ARAT) and group allocation data for study participants are summarized in Table 1 (comorbidities are reported in Supplementary Table 2).

**Table 1:** Randomized feasibility trial: participants’ characteristics

	Age (years)	Sex	Stroke type	Months since stroke	Impaired arm	FMA-UE (0−66)	ARAT (0−57)	Barthel Index (0−100)	Kinematic baseline data (mean velocity, m s^−1^)	Group
P01	74	M	Ischemic	216	Right	44	37	85	0.3472	MS
P02	65	M	Ischemic	23	Right	45	40	95	0.1965	MS
P03	47	M	Hemorragic	3	Left	41	28	95	0.2665	MS
P04	44	M	Ischemic	129	Right	33	32	70	0.1285	MS
P05	54	M	Ischemic	22	Right	40	40	90	0.1023	MS
P06	62	M	Ischemic	13	Right	51	55	95	0.1815	MS
P07	47	F	Ischemic	3	Left	13	0	75	0.2157	MS
P08	46	F	Ischemic	18	Left	21	3	90	0.1546	MS
P09	63	F	Ischemic	12	Left	51	52	90	0.1028	MS
P10	62	M	Ischemic	12	Left	23	3	80	0.1062	MS
P11	62	F	Ischemic	23	Left	35	31	80	0.2759	MS
P12	60	M	Ischemic	11	Right	42	53	85	0.2319	MS
PC01	42	F	Ischemic	6	Right	43	38	90	-	C
PC02	67	M	Ischemic	12	Right	53	57	90	-	C
PC03	62	M	Ischemic	11	Left	16	0	80	-	C
PC04	57	M	Ischemic	8	Left	50	29	95	-	C
PC05	49	M	Ischemic	12	Left	60	57	85	0.3254	C
PC06	78	F	Ischemic	9	Left	56	57	95	0.1853	C
PC07	47	F	Ischemic	3	Left	29	20	75	0.1340	C
PC08	77	M	Ischemic	287	Right	15	0	95	0.1630	C
PC09	63	M	Ischemic	162	Left	34	36	90	0.2360	C
PC10	52	M	Ischemic	20	Left	10	3	65	0.1767	C
PC11	67	F	Ischemic	19	Right	49	21	55	0.0639	C
PC12	68	F	Ischemic	6	Left	43	43	95	0.0657	C
PC13	62	M	Ischemic	3	Left	53	57	85	0.2724	C

C, conventional rehabilitation group; F, female; M, male; MS, MultiSensy group.

Statistical analyses between the two groups were performed to ensure comparability regarding age, months since stroke and FMA-UE and ARAT scores (Supplementary Table 3).

### MultiSensy platform and pilot results

**Fig. 2:**
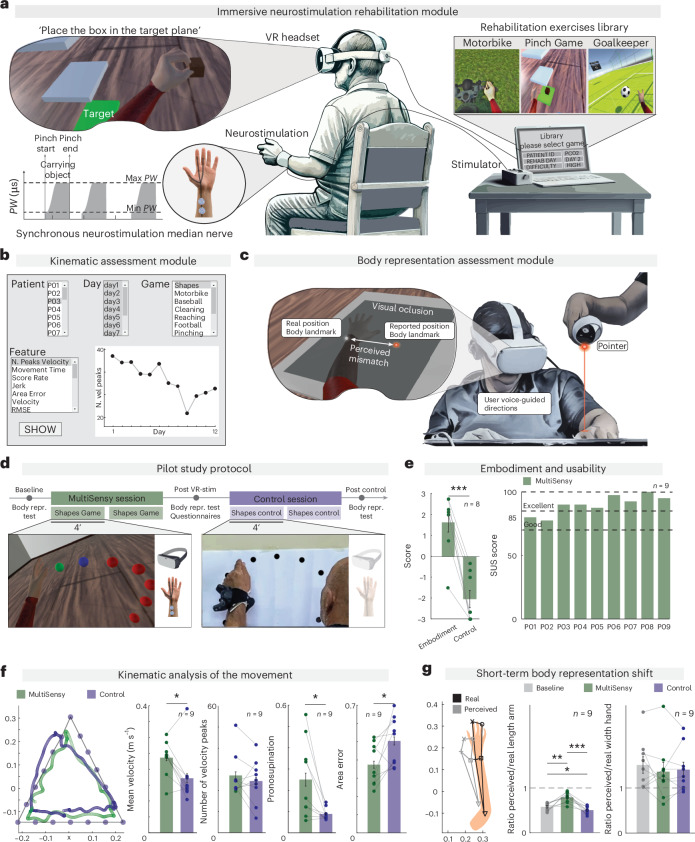
MultiSensy: immersive neurostimulation platform for stroke survivors. **a**, Immersive neurostimulation rehabilitation module. Participants are immersed in VR and receive TENS targeting the median nerve (impaired arm) with pulse-width modulation depending on the task. Left, an example of task: Pinch Game. TENS is triggered upon the object pinching; it is modulated to reach its maximum following Gaussian-like profile and to stop upon releasing the object. Right, a display of customizable upper-limb rehabilitation scenarios. **b**, Kinematic assessment module. The kinematic GUI allows to track and visualize participants’ progress over time for specific features. **c**, Body representation assessment module. During the Body Landmark Test execution, the participant places their impaired hand on a table, which remains visually occluded in VR. Using vocal commands, they guide a laser pointer to align with the perceived position of specific hand landmarks. **d**, Pilot study protocol. Patients completed task-oriented sessions under two conditions: MultiSensy and control. In MultiSensy, patients perform two consecutive rounds (4 minutes each) of the Shapes Game. In control, patients replicate the same movements without VR and TENS, using wall-mounted posters matching the virtual shapes. Baseline and post-condition assessment included the Body Landmark Test. Usability (SUS) and embodiment questionnaires were collected after the MultiSensy condition. **e**, Embodiment and usability. Mean ± s.e.m. embodiment and control scores for *n* = 8 participants are displayed as bar plots (two-sided paired *t*-test, *P* = 0.0001). Individual SUS scores are shown on the right. **f**, Kinematic analysis. On the left, an example hand trajectory from the Shapes Game. On the right, mean ± s.e.m. values for kinematic indices—mean velocity (*P* = 0.025), number of velocity peaks (*P* = 0.409), pronation−supination range (*P* = 0.021) and area error (*P* = 0.034)—are displayed for *n* = 9 participants under the two experimental conditions (two-sided paired *t*-test). **g**, Short-term body representation shift. On the left, a graphical representation of the real (black, aligned with the forearm) and perceived (grey) positions of the five landmarks of interest. On the right, the mean ± s.e.m. ratios (*n* = 9) of perceived to real arm length (*P*_MultiSensy_baseline = 0.0037, *P*_baseline_control = 0.0341, *P*_MultiSensy_control = 0.0004) and hand width are shown for each condition (repeated-measures ANOVA with two-sided LSD post hoc). *P < 0.05, **P < 0.01, ***P < 0.001. 4′, 4 minutes; Max, maximum; Min, minimum; repr., representation; stim, stimulation.

MultiSensy is an immersive neurostimulation platform for stroke survivors. It integrates custom immersive VR scenarios with congruent transcutaneous neural stimulation of the median nerve for sensory restoration to offer personalized stroke rehabilitation and assessment (Fig. 2).

On the rehabilitation side, the platform features nine personalized rehabilitation games, each designed to target specific movement and muscular functions (Fig. 2a). The games require the use of the impaired arm exclusively to repetitively perform task-oriented movements, as inspired by constraint-induced therapy (Fig. 2a). The performed movements are combined with task-specific, congruent sensory neurostimulation targeting the median nerve. The electrical stimulation is calibrated to transcutaneously activate sensory fibers in the nerve innervating the thenar region and palmar territories of the thumb, index and middle fingers, which are directly involved in object manipulation during the VR tasks (Fig. 2a). On the assessment side, the platform continuously captures hand and arm poses at a frequency of 50 Hz, generating motion data and kinematic indicators (Fig. 2b). Beyond kinematic assessments, the platform includes two dedicated scenarios for evaluating body perception: the Body Landmark Test^60^ (Fig. 2c) and the PPS task^20^ (see ‘Body representation assessment’ section).

The pilot study involved a single-day session where participants performed a shape-tracking task on a vertical plane under two conditions. In the first (MultiSensy), they completed the task within the immersive neurorehabilitation platform (Shapes Game; see ‘Rehabilitation games’ section). In the second, they replicated the task in a real-world scenario using a wall-mounted poster with identical shapes (control) (Fig. 2d). Both conditions lasted 8 minutes, and their order was randomized across participants. Participants evaluated the platform experience with an embodiment (Supplementary Table 4)^47,61^ and a System Usability Scale (SUS) (Supplementary Table 5)^62^ questionnaire. Embodiment and control scores (each ranging from −3 to +3) were statistically compared to confirm that participants experienced stronger embodiment sensations than those reflected by the control items^63^. Participants felt embodied in the platform: embodiment score = 1.64 ± 0.50 (mean ± s.e.m.), control score = −2.04 ± 0.41 (two-sided paired *t*-test, *P* < 0.001, Cohenʼs *d* = 2.64) (Fig. 2e). Participants could intuitively use the platform, with seven of nine rating its usability as ‘excellent’ (SUS > 80.8) and the remaining two as ‘good’ (SUS > 71.1)^64^ (Fig. 2e). Kinematic analysis of the movements performed in the two conditions is shown in Fig. 2f, highlighting representative kinematic indices (full list in Supplementary Table 6). Velocity (MultiSensy = 0.24 ± 0.02 (m s^−1^, mean ± s.e.m.), control = 0.17 ± 0.03, *P* = 0.025) and pronosupination range (MultiSensy = 0.25 ± 0.06, control = 0.09 ± 0.01, *P* = 0.021) were higher, whereas area error (MultiSensy = 0.37 ± 0.04, control = 0.5 ± 0.04, *P* = 0.034) was lower in the immersive neurostimulation platform compared to the control condition (Fig. 2f). The smoothness of movement, measured by the number of velocity peaks, showed no significant difference between the two conditions (Fig. 2f). The Body Landmark Test was conducted first at baseline and each time after every experimental condition. The arm length ratio (between perceived and real dimension) improved after the MultiSensy condition (MultiSensy = 0.783 ± 0.039, mean ± s.e.m.) compared to both the baseline (0.571 ± 0.026, *P* < 0.001) and control (0.499 ± 0.026, *P* = 0.0004) conditions (Fig. 2g). No significant differences were observed for the hand width ratio (Fig. 2g). Additional indexes and the full statistical analysis are provided in Supplementary Table 7.

### Primary outcomes

**Fig. 3:**
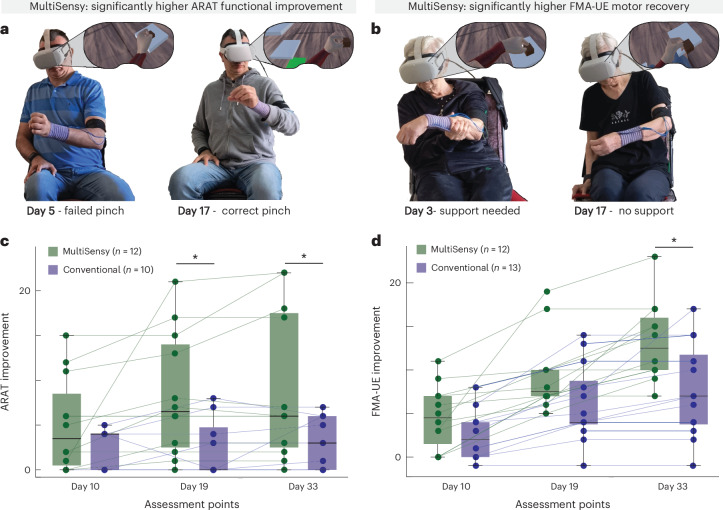
ARAT and FMA-UE clinical improvements. **a**, ARAT patient improvement—visual example. On day 5 (session 4), P03 fails to execute a correct pinch but successfully performs it on day 17 (session 11). **b**, FMA-UE patient improvement—visual example. On day 3 (session 2), P07 requires support from the unimpaired arm to complete the movement, whereas, on day 17, (session 11), they perform it independently. **c**,**d**, Improvements in ARAT (**c**) and FMA-UE (**d**) scores are shown in T1, T2 and T3 for the MultiSensy (green, *n* = 12) and conventional rehabilitation (blue, *n* = 13) groups. Participants with ARAT = 57 at T0 were excluded from this analysis. Box plots depict the median, interquartile range (IQR) and whiskers extending to the most extreme data points within 1.5× IQR. Individual data points are overlaid on each box plot. Two-sided independent *t*-tests were used for statistical comparisons (ARAT_T2 *P* = 0.029, ARAT_T3 *P* = 0.049, FMA-UE_T3 *P* = 0.01). *P < 0.05, **P < 0.01, ***P < 0.001.

The primary outcomes of the randomized feasibility trial included motor function assessed by the ARAT and FMA-UE and self body representation assessed by the Body Landmark Test. Single-subject analysis revealed that, in the MultiSensy group, eight of 12 patients achieved a clinically meaningful ARAT improvement (>5.7 (ref. ^29^)) by T3 compared to three of nine (the other four patients already had 57/57 score) in the conventional rehabilitation group (Fig. 3a,c and Extended Data Table 3).

We next applied a linear mixed-effects model with fixed effects of Group (MultiSensy versus Conventional), Time (T0–T3) and their interaction and random intercepts for participants. The reference levels were Group = Conventional and Time = T0. The mixed model yielded a significant Group × Time interaction (*F*_3,74_ = 3.81, *P* = 0.013), whereas the main effects of Time (*F*_3,74_ = 1.49, *P* = 0.224) and Group (*β* = 10.06, 95% confidence interval: −5.31 to 25.42, *P* = 0.196) were not significant. Post hoc comparisons showed significantly greater ARAT improvements in the MultiSensy group at both T2 (MultiSensy = 8.25 ± 1.96, Conventional = 2.44 ± 1.08, *P* = 0.029) and T3 (MultiSensy = 9.17 ± 2.37, Conventional = 3.11 ± 0.98, *P* = 0.049) relative to the Conventional group (Fig. 3c and Supplementary Table 8).

For FMA-UE, all 12 patients in the MultiSensy group achieved a clinically meaningful improvement (>5.25 (ref. ^65^)) by T3, whereas eight of 13 patients in the conventional rehabilitation group showed similar progress (Fig. 3b,d and Extended Data Table 3). The linear mixed-effects model revealed a significant main effect of Time (*F*_3,89_ = 20.50, *P* < 0.001) and a significant Group × Time interaction (*F*_3,89_ = 4.86, *P* = 0.0035), indicating that motor recovery trajectories differed between groups over time. The Group main effect (baseline difference) was not significant (*β* = −2.72, 95% confidence interval: −14.08 to 8.63, *P* = 0.635). Post hoc comparisons of baseline-corrected change scores confirmed significantly greater improvements in the MultiSensy group at T3 (MultiSensy = 13.17 ± 1.30, Conventional = 7.54 ± 1.48, *P* = 0.01) (Fig. 3d and Supplementary Table 8).

**Fig. 4:**
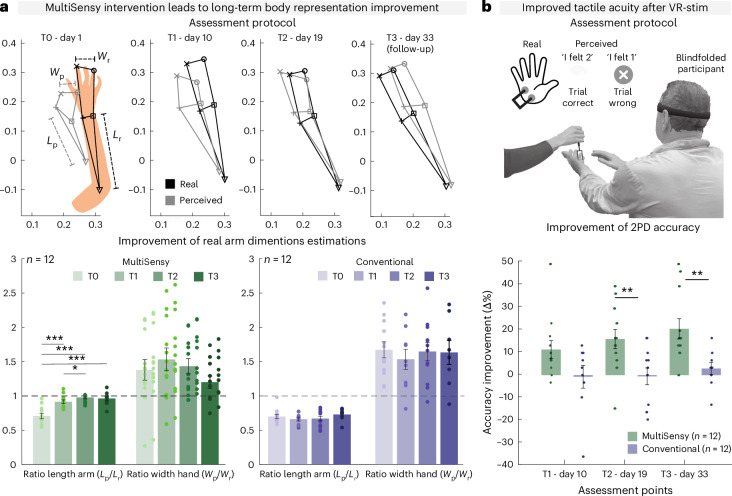
Body representation and sensory improvements. **a**, MultiSensy intervention leads to sustained body representation improvements. Top, example data from participant P01 showing real (black, aligned with the forearm) and perceived (gray) positions of five landmarks at T0, T1, T2 and T3. Arm length (*L*) and hand width (*W*) are displayed in dashed lines. Bottom, mean ± s.e.m. ratios of perceived to real arm length and hand width are shown for each assessment point for the MultiSensy (green, *n* = 12) and conventional rehabilitation (blue, *n* = 12) groups. Repeated-measures ANOVA with two-sided LSD post hoc analysis was used (T0 versus T1, T0 versus T2 and T0 versus T3; all *P* < 0.001). The bar plots show that only participants in the MultiSensy group realigned their distorted body perception (shorter arm length and wider hand width) toward actual body dimensions (ratio = 1). **b**, Improved tactile acuity after MultiSensy. Blindfolded participants receive repeated touches on the palm of the impaired hand with either one or two pins. They report the number of pins they perceive. A response is considered correct when the perceived and actual number of pins match. Bottom, mean ± s.e.m. improvements in 2PD (tactile acuity) scores at T1, T2 (*P* = 0.008) and T3 (*P* = 0.006) are shown for the MultiSensy (green, *n* = 12) and conventional rehabilitation (blue, *n* = 12) groups (two-sided independent *t*-test). *P < 0.05, **P < 0.01, ***P < 0.001. p, perceived; r, real; stim, stimulation.

For the Body Landmark Test, participants in the MultiSensy group demonstrated improved arm length ratio perception at day 10, T1 (T0 = 0.709 ± 0.039 (mean ± s.e.m.), T1 = 0.916 ± 0.028, *P* < 0.001), with further improvements at day 19, T2 (T2 = 0.979 ± 0.021, *P* versus T1 = 0.02) and sustained at day 33, T3 follow-up (0.965 ± 0.027, *P* versus T0 < 0.001) (Fig. 4a and Extended Data Table 4).

By contrast, no significant improvements were observed for hand perception. Participants in the conventional rehabilitation group showed no improvements in body representation at either the arm or hand level (Fig. 4a and Extended Data Table 5).

### Secondary outcomes

Secondary outcomes of the randomized feasibility study included functional independence in ADLs, sensory acuity and PPS. For the Barthel Index, measuring functional independence, four of 12 patients in the MultiSensy group and two of 13 patients in the conventional rehabilitation group showed clinically meaningful improvement by T3 (Extended Data Table 3). However, the linear mixed-effect model revealed no Group × Time interaction (*P* > 0.05). Participants’ sensory acuity was assessed using the 2-point discrimination (2PD) tactile acuity test (Fig. 4b). At each assessment point, participants were touched repeatedly (*n* = 40) with either one or two pins and asked to report the number of pins they felt. Accuracy in responses (0−1, where 1 represents 100% accuracy) was used as the tactile acuity outcome (Fig. 4b). Between-group analysis revealed significantly higher 2PD performance improvements at T2 (MultiSensy = 0.162 ± 0.044 (mean ± s.e.m.), conventional = −0.006 ± 0.038, *P* = 0.008) and T3 (MultiSensy = 0.208 ± 0.046, conventional = 0.030 ± 0.028, *P* = 0.006) in the MultiSensy group compared to the conventional rehabilitation group (Fig. 4b and Supplementary Table 9). For PPS, reaction times across assessment points and groups are shown in Supplementary Fig. 1. To evaluate PPS changes, a two-way (distances × assessment points) within-subjects ANOVA was conducted. For both groups, the ANOVA revealed no significant interaction effects (MultiSensy *P* = 0.23, conventional *P* = 0.37), indicating no multisensory facilitation (reduction in reaction times) at different distances across timepoints.

### Safety

No adverse events were reported during either the pilot study or the randomized feasibility study.

### Exploratory outcomes

**Fig. 5:**
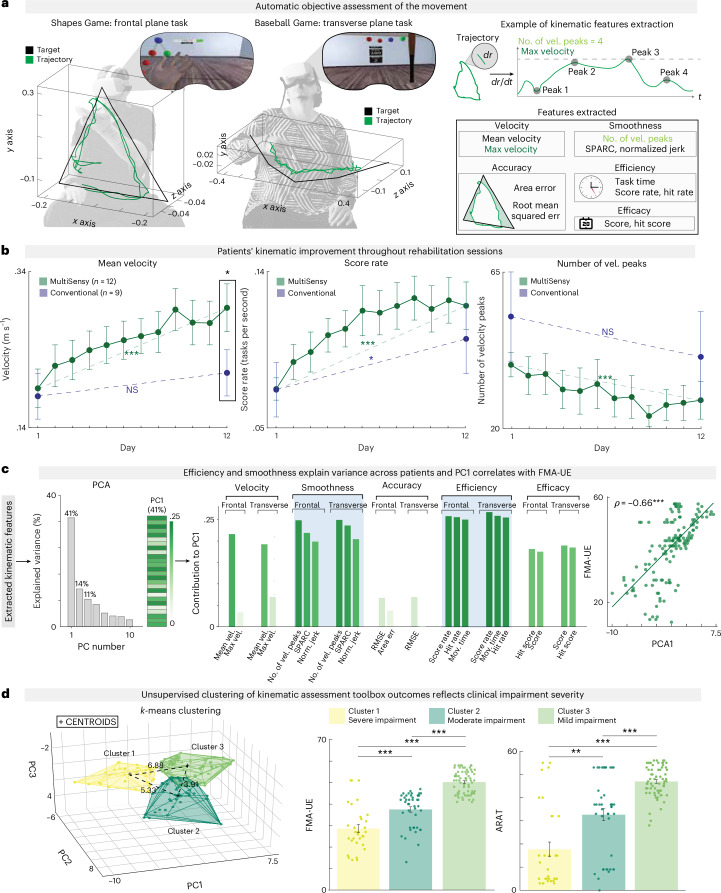
MultiSensy objective assessment module of patients’ performances. **a**, Automatic objective assessment of the movement. Left, representative movement trajectories during frontal (Shapes Game) and transverse (Baseball Game) plane tasks. Green: actual trajectory; black: target trajectory. Right, extraction of kinematic features from trajectory and velocity profiles. Extracted features included velocity, smoothness, accuracy, efficiency and efficacy indicators. **b**, Patients’ kinematic improvement throughout rehabilitation sessions. Shapes Game mean ± s.e.m. values for velocity, score rate and smoothness (number of velocity peaks) are shown for the MultiSensy group (*n* = 12) across 12 sessions and the conventional rehabilitation group (*n* = 9) for sessions 1 and 12. Repeated-measures ANOVA with two-sided LSD post hoc was used for within-group comparisons in MultiSensy, with significance marks for intervention session 1 versus intervention session 12 displayed (velocity, score rate and number of velocity peaks; all *P* < 0.001). Two-sided paired *t*-tests were used for within-group comparisons in the conventional rehabilitation group (intervention session 1 versus intervention session 12, score rate *P* = 0.03), and two-sided independent *t*-tests assessed between-group improvement (intervention session 12 − intervention session 1, velocity *P* = 0.012). **c**, Efficiency and smoothness explain variance across patients, and PC1 correlates with FMA-UE. Left, variance explained by PCA and feature loadings of PC1. Center, PC1 loadings grouped by indicator type (velocity, smoothness, accuracy, efficiency and efficacy) and task (frontal and transverse). Right, correlation between PC1 and FMA-UE clinical scores. Pearsonʼs correlation analysis was performed. **d**, Unsupervised clustering of kinematic assessment toolbox outcomes reflects clinical impairment severity. Left, the first eight PCs were used to derive three *k*-means clusters. The plot includes 143 points (one per patient session). The clusters are visualized on the first three PCs. Right, based on their associated clinical score distributions, clusters 1, 2 and 3 correspond to severe, moderate and mild impairment, respectively. FMA-UE and ARAT scores are shown as mean ± s.e.m. for each cluster, with individual points representing interpolated session scores. One-way ANOVA with two-sided LSD post hoc tests showed significant differences between all clusters for FMA-UE and ARAT (*P* < 0.001 except cluster 1 versus cluster 2 at *P* = 0.0013). *P < 0.05, **P < 0.01, ***P < 0.001. *dr*, differential of displacement; *dt*, differential of time; err, error; Max, maximum; Mov., movement; Norm., normalized; NS, not significant; PC, principal component; RMSE, root mean squared error; SPARC, Spectral Arc Length; vel., velocity.

During the rehabilitation games in the immersive neurostimulation platform, we extracted kinematic indicators capturing movement velocity, smoothness and accuracy as well as performance indexes quantifying efficiency and efficacy^59^ (Fig. 5a and Supplementary Table 10).

MultiSensy games were selected individually by physiotherapists, but two (Shapes and Baseball games) were common to most participants to enable cross-participant comparisons. The Shapes Game involved repetitive hand movements in the frontal plane, and the Baseball Game required hand movements in the transverse plane. Because the conventional rehabilitation group did not use the platform during the trial, its participants completed two sessions of the Shapes Game (frontal plane task), without stimulation, once on the first and once on the last rehabilitation day. This allowed assessment of their overall kinematic improvements during their conventional rehabilitation in the randomized feasibility study.

Representative kinematic indexes from the frontal plane task are shown in Fig. 5b. In the MultiSensy group, participants significantly improved velocity (repeated-measures ANOVA *P* < 0.001, intervention 1 = 0.19 ± 0.03 (mean ± s.e.m.), intervention 12 = 0.29 ± 0.03, *P* < 0.001), score rate (repeated-measures ANOVA *P* < 0.001, intervention 1 = 0.07 ± 0.01, intervention 12 = 0.12 ± 0.02, *P* < 0.001) and smoothness (repeated-measures ANOVA *P* < 0.001, intervention 1 = 38.32 ± 3.47, intervention 12 = 28.17 ± 5.44, *P* < 0.001) throughout the rehabilitation sessions (Fig. 5b). In the conventional rehabilitation group, improvements in velocity (intervention 1 = 0.18 ± 0.03, intervention 12 = 0.21 ± 0.03, *P* = 0.09) and smoothness (intervention 1 = 52.17 ± 12.80, intervention 12 = 40.61 ± 12.93, *P* = 0.3) were not significant, whereas score rate increased significantly from intervention 1 to intervention 12 (intervention 1 = 0.07 ± 0.02, intervention 12 = 0.10 ± 0.02, *P* = 0.03) (Fig. 5b). Between-group analysis revealed significantly higher velocity improvement in the MultiSensy group (*P* = 0.011) compared to the conventional rehabilitation group (Fig. 5b).

To explore variance in task performances and to investigate the clinical relevance of kinematic indicators, we applied unsupervised machine learning to data from the frontal and transverse plane tasks for the MultiSensy group. Principal component analysis (PCA) revealed that the first principal component (PC1) accounted for 41% of the total variance, and the first eight components together explained 90% of the total variance (Fig. 5c). The weights of PC1 were analyzed to identify the kinematic features that contributed the most to variability in these tasks. In both tasks, features related to efficiency (success rate, task time and hit rate) and smoothness (number of velocity peaks) emerged as key drivers of variance. Notably, none of the features associated with accuracy, speed or efficacy ranked among the top contributors to PC1. PC1 showed a significant correlation with clinical FMA-UE (Pearsonʼs *ρ* = 0.66, *P* < 0.001) (Fig. 5c) and ARAT (*ρ* = 0.60, *P* < 0.001) scores.

The first eight principal components were further used for *k*-means clustering, associated with distinct levels of clinical impairment severity (Fig. 5d). Based on their associated clinical score distributions, cluster 1, cluster 2 and cluster 3 corresponded to severe, moderate and mild impairment, respectively. Indeed, patients’ clusters referred to significantly different FMA-UE scores (cluster 1: 28.59 ± 1.84, cluster 2: 37.49 ± 1.24, cluster 3: 50.24 ± 0.64; ANOVA *P* < 0.001, all post hoc *P* < 0.001) and ARAT scores (cluster 1: 17.72 ± 3.12, cluster 2: 32.63 ± 2.61, cluster 3: 46.97 ± 0.79; ANOVA *P* < 0.001, all post hoc *P* < 0.01) (Fig. 5d). Notably, the differences in mean FMA-UE and ARAT scores across clusters were not only statistically significant but also exceeded the minimal clinically important difference (MCID) for both FMA-UE (5.25 (ref. ^65^)) and ARAT (5.7 (refs. ^29,66^)), underscoring their clinical relevance.

## Discussion

In this study, we introduced MultiSensy: a multimodal immersive neuro-platform for personalized stroke rehabilitation and assessment. We first conducted a pilot study with nine stroke survivors, demonstrating the platform’s usability and short-term benefits. This was followed by a randomized feasibility study involving 25 patients with chronic stroke, which showed that MultiSensy outperformed conventional physiotherapy across a range of stroke-induced impairments.

### Platform usability and short-term effects

Results from the pilot study demonstrated excellent usability, with seven of nine participants reporting high SUS scores, highlighting the platform’s user-friendly, robust and intuitive design. These results are particularly notable, as technical and design-related challenges can hinder the adoption of immersive technologies, particularly among older patients, thereby limiting their rehabilitation potential^67,68^. The strong sense of embodiment with the virtual avatar further reinforced the ability of the platform to engage users and create an immersive environment. This effect can be attributed to the first-person perspective, the precise mirroring of avatar movements with the patients’ actions and the synchrony between visuomotor interactions and tactile stimulation^50,52,69,70^. In this pilot trial, participants exhibited improved kinematics in the task performance, with higher velocity and greater forearm pronosupination, likely driven by the platform’s immersive design fostering engagement and motivation^71^. Pronosupination gains may be attributed to the targeted neurostimulation feature of the game, which encouraged participants to orient the palm toward virtual targets to receive a congruent tactile feedback, thereby promoting repeated forearm rotation, a crucial movement for rehabilitation^72^. In the Body Landmark Test assessment, our baseline findings aligned with previous literature, confirming that patients with stroke misperceive their impaired arm as shorter^15,18^. Notably, we observed short-term improvements in arm representation after completion of the task. Although the rehabilitation task duration (8 minutes) exceeded the threshold for inducing body illusions^73,74^, the debated duration of such effects after the illusion prevents us from conclusively attributing the improvement solely to this factor. Therefore, these results prompted a longitudinal analysis in the planned randomized feasibility study.

### Clinical effectiveness of the platform

In the randomized feasibility study, the neuro-immersive intervention demonstrated clinically meaningful improvements at follow-up in ARAT and FMA-UE scores for eight of 12 and for 12 of 12 patients, respectively. Clinically meaningful improvements were determined based on the MCID, defined as an improvement of 5.7 points for ARAT and of 5.25 points for FMA-UE, commonly used as responder endpoints in stroke clinical trials.

The observed improvements at the end of the MultiSensy intervention (T2: ΔARAT > 8, ΔFMA-UE > 9; T3: ΔARAT > 9, ΔFMA-UE > 13) exceeded those reported in most studies on chronic patients using electrical stimulation platforms^18,75^ or VR-based approaches^76–82^ yet remained within the range of a few studies^83–85^, positioning our results among the highest reported for similar interventions. Notably, these outcomes were instead similar to those achieved with immersive VR in subacute patients^85–87^ who are prone to show greater recovery^5^. When compared to previous FES-based and robotics-based rehabilitation studies, the improvements achieved in the MultiSensy group ranked at the upper end of outcomes reported for hybrid FES−robotic intervention in chronic stroke^30,88^. Studies combining robotic therapy with electromyography (EMG)-triggered stimulation in subacute patients have shown similar or smaller effects^89,90^. This suggests that the integration of synchronous multisensory feedback within the immersive MultiSensy protocol may potentiate neuroplastic mechanisms and functional recovery beyond what is typically achieved with conventional robotic or FES-based approaches, even in chronic stroke survivors.

Improvements were also observed in the conventional rehabilitation group, with three and eight participants achieving MCID improvements in ARAT and FMA-UE, respectively. Both groups demonstrated consistent gains in upper-limb motor and functional scores as early as T1, after six rehabilitation sessions. These results likely reflect the often underutilized motor and functional potential in this population, attributed to ‘learned non-use’^91,92^ of the paretic arm due to prolonged lack of confidence, motivation and access to adequate chronic rehabilitation. As a result, chronic patients may underperform in assessments, not due to irreversible deficits but because they habitually avoid using the affected limb. However, targeted rehabilitation, even over a short period, can rapidly restore this underutilized potential, explaining the early improvements observed in both groups. After evaluating within-group variations in clinical scales, we conducted between-group comparisons, demonstrating that the neuro-platform significantly outperformed conventional rehabilitation. Improvements in ARAT were already significant on day 19 (T2) and sustained on day 33 (T3), whereas FMA-UE improvements reached significance on day 33 (T3). The findings suggest that the developed neuro-platform, which requires minimal supervision during task execution, could match or even surpass the effectiveness of conventional rehabilitation. Conventional approaches often rely heavily on physiotherapy resources, in terms of both personnel and time, making the neuro-platform a potentially more scalable and efficient solution for chronic stroke recovery.

One possible explanation for the greater efficacy observed in the MultiSensy group lies in the role of motivation and reward-based engagement. Unlike conventional therapy, the immersive environment of the platform offered patients real-time, success-contingent feedback and emotionally engaging, goal-directed tasks, conditions known to facilitate reinforcement learning^35,36^. Motivation and reward are not just psychological bonuses in rehabilitation but, also, biological drivers of motor recovery^93,94^. These elements are known to engage the brain’s reward system, notably the lateral hypothalamus and dorsal raphe nucleus, which modulate dopaminergic activity in the ventral tegmental area ^37,38,95,96^. The ventral tegmental area projects to the primary motor cortex, where dopamine release can induce neuroplastic changes essential for motor learning^39,40^. In our intervention, such reward-driven neuromodulation may have supported the consolidation of motor patterns, contributing to the sustained improvements observed in the MultiSensy group.

### Sensory and body representation improvements

Diverse evidence documents sensory impairments after a stroke^8,97,98^, with tactile acuity frequently employed to assess it^18,99^. Our findings showed significantly greater improvements in tactile acuity (2PD test) for the MultiSensy group compared to conventional rehabilitation. This outcome is likely reflected in both peripheral and central mechanisms. Although peripheral pathways remain intact in patients with stroke, targeted sensory neurostimulation may still be beneficial by retraining sensory fibers that have become underactive due to patients’ habitual avoidance of using the paretic limb, supporting confidence in their movement. At the same time, sensory neurostimulation may target sensory nerves projecting to brain-damaged areas, hence activating specific neural pathways and promoting functional reorganization^100–102^. On a central level indeed, 2PD is strongly linked to body representation, with clinical evidence showing that changes in somatosensory cortex representation fields are tied to altered thresholds^103–107^. In this view, these results may then suggest neuroplastic changes induced by the neuro-immersive intervention toward a restored body representation.

This central perspective is further supported by the Body Landmark Test results, which demonstrated improvements in arm representation after just six rehabilitation sessions, further enhanced by the end of the intervention and maintained at the 2-week follow-up. Unlike the pilot phase, these improvements cannot be attributed to short-term VR immersion, as assessments occurred on separate days (2 weeks) from the rehabilitation sessions. Instead, they reflect sustained enhancements in body perception driven by the multimodal intervention. These findings represent a new quantitative perspective on the restorative potential of body ownership illusions in addressing stroke-induced body representation deficits. These deficits, including misjudged body position^15^, phantom limb sensations^17^ and anosognosia^108^ (denial of sensory-motor deficits), have been extensively studied even in other populations^109^. However, clinical evidence linking improved body representation to multimodal interventions modulating body illusions has been lacking^53^. Our results address this critical gap, offering evidence that could guide the development of innovative rehabilitation strategies.

### Kinematic indicators of recovery

Kinematic analysis showed substantial improvements across most kinematic indexes in the MultiSensy group throughout the rehabilitation period. By contrast, the conventional rehabilitation group showed inconsistent improvements, emphasizing the platform’s greater impact on enhancing task-specific movement execution. These results likely stem from the personalized, task-oriented design of the immersive platform, which enhances engagement and facilitates motor learning. However, we cannot exclude the contribution of habituation effects to the virtual environment. Indeed, participants in the Multisensy group interacted with the platform across multiple sessions, compared to the conventional group, which engaged with it only on the first and last rehabilitation days.

Beyond these findings, it is crucial to recognize the importance of kinematic analysis in complementing clinical scales. Unlike traditional assessments, which are resource intensive, reliant on trained personnel and suffer from inter-examiner variability, kinematic indexes offer an intrinsic advantage: they capture motor performance directly during task execution^59,110^. This allows real-time, objective monitoring of patient status without disrupting the rehabilitation workflow.

To gather more insight into the extracted kinematic indexes, we performed a PCA on data from the Shapes and Baseball games, which involve repeated movements in the frontal and transverse planes, respectively. The analysis revealed that efficiency and smoothness were the main contributors to inter-participant variability, highlighting their importance in assessing movement quality and differentiating motor performance. These indexes thus emerge as sensitive indicators of motor recovery, providing clinicians with a valuable tool to track patient progress. To establish their clinical validity, we demonstrated significant correlations between the principal components and standard scales (FMA-UE and ARAT), confirming the potential of kinematic metrics as reliable markers of functional status. Moreover, the principal components enabled differentiation between clinically distinct patient clusters, further reinforcing their diagnostic value.

This finding suggests that the platform can infer motor and functional status directly from gameplay data, eliminating the need for frequent, time-intensive clinical evaluations. This capability not only validates the clinical relevance of kinematic assessments but also lays the foundation for future adaptive brain−computer interface (BCI) systems, which could leverage real-time kinematic data to dynamically tailor rehabilitation protocols, enabling fully personalized, home-based, data-driven therapy.

### Limitations and future perspectives

This study is a randomized feasibility study serving as a proof-of-concept investigation of a novel therapy. Its efficacy has yet to be fully validated in larger clinical trials. At its current stage, it presents some limitations. First, the follow-up period was limited to 2 weeks, providing only partial insight into the persistence of the observed effects. Hence, extending the follow-up duration will be an objective in future trials. Moreover, although we can exclude differences in rehabilitation as both conditions were matched for therapy duration and exercise content, it remains unclear whether the observed clinical and body representation MultiSensy improvements were primarily driven by the VR-based games, the targeted sensory neurostimulation or their combination. In the present study, we were motivated by targeting both sensorimotor components together; future studies could potentially incorporate single-component conditions (for example, VR-only or sensory neurostimulation-only) to isolate and evaluate the contribution of each modality to the intervention’s outcomes. Regarding the assessments, we acknowledge the presence of occasional missing data from some participants as a limitation. These data gaps arose from practical and logistical constraints inherent to an active inpatient clinical setting. In future larger-scale studies, improved scheduling, staffing and resource allocation will be implemented to further reduce missing data. Overall, although the setup is inherently suitable for home-based rehabilitation, the current trial was conducted in clinical settings. This limits our findings to an initial indication of the platform’s potential for fully remote rehabilitation programs. Follow-up trials will focus on evaluating its effectiveness and feasibility in home-based environments.

## Conclusion

Our results show that a digital health platform combining immersive reality and targeted sensory neurostimulation leads to clinically meaningful improvements in motor function, sensory acuity and body representation in patients with chronic stroke. The system enables real-time, objective monitoring of rehabilitation progress through automated kinematic analysis. Building upon this, we envision next-generation digital therapies that are accessible, personalized and scalable for at-home neurorehabilitation.

## Methods

### Participants

Participants were recruited through the Clinic for Rehabilitation ‘Dr. Miroslav Zotovic’ in Belgrade, Serbia. A total of 34 patients with stroke participated in the study. Nine patients participated in the pilot phase of the study, and 25 patients participated in the randomized feasibility study (see CONSORT diagram; Fig. 1). Biological sex was recorded based on participant self-report. Gender identity was not collected. Participant age and sex are reported in Table 1 and Extended Data Table 2. The study was not designed to assess sex-specific or gender-specific effects; therefore, no sex-based or gender-based subgroup analyses were performed. Participants did not receive financial compensation for participation. Inclusion and exclusion criteria are specified in Extended Data Table 1. All participants read and signed the informed consent form. The experiments were designed and conducted in accordance with the Declaration of Helsinki and received approval from the Ethical Committee of the Clinic for Rehabilitation ‘Dr. Miroslav Zotovic’ (numbers 03-2105/1 and 03-788/2). The randomized feasibility study was registered with ClinicalTrials.gov (NCT06400823).

### Multisensory platform

The multisensory platform integrates a wearable immersive reality headset (Meta Quest 2; Meta) with an electrical stimulator (RehaMove3; HASOMED) that delivers 50-Hz biphasic pulses targeting the median nerve (Fig. 2a). For the pilot phase only, we used a VIVE Pro (HTC) headset instead of the Meta Quest 2, equipped with three VIVE trackers positioned on the elbow and wrist of the impaired arm and on the hand of the unimpaired arm. The software platform, developed using Unity, leverages custom C# scripts to create immersive rehabilitation and assessment scenarios and seamlessly controls the electrical stimulator through a custom-developed pipe server, enabling precise stimulation modulation. The system provides a fully customizable library for stroke rehabilitation and assessment, tailored to the specific needs of each patient (Fig. 2a). In all scenarios, participants are fully immersed in a first-person perspective, embodying an avatar. In the pilot phase, hand recognition was achieved using VIVE trackers attached to participants’ limbs to capture movements. For the randomized feasibility study phase, markerless Meta Quest 2 hand recognition modules were seamlessly integrated with custom-developed inverse kinematics to animate the avatar’s shape and pose, precisely mirroring the participant’s movements^111^. The rehabilitation component of the platform includes nine personalized rehabilitation games, each designed to target specific movement and muscular functions (Fig. 2a). Before performing each game, participants view instructional videos demonstrating the required movements and objectives to ensure proper task execution. The games require the use of the impaired arm exclusively, simulating a virtual constraint-induced therapy setup by prohibiting task completion with the unimpaired hand. Participants interact with the virtual environment through task-oriented movements, completing specific objectives designed to enhance motor recovery (Fig. 2a). The performed movements are complemented by synchronous, targeted TENS of the median nerve in the impaired hand (Fig. 2a). The stimulation is task specific and simulates the interaction of the virtual ‘impaired’ hand with virtual objects. It spreads across the palm and fingers, delivering additional sensory input that enhances the feedback provided by the virtual environment. The intensity of the electrical stimulation is calibrated individually during an initial setup phase. While performing the tasks, participants always see a small black panel displaying their current score, enhancing engagement and motivation to improve their performance. The platform’s assessment component operates both passively and actively. During task performance, it seamlessly captures the pose of the participant’s hands and arms at a refresh rate of 50 Hz, providing continuous motion data (Fig. 2b). In addition to this embedded assessment, the platform includes dedicated scenarios specifically designed to evaluate participant body perception: a VR-based adaptation of the Body Landmark Test^60^ (Fig. 2c) and a visuo-tactile PPS^112^ task.

### Rehabilitation games

The game design process was clinician driven, based on observations of rehabilitation sessions and therapist inputs to identify key movement patterns and functional tasks. Therapists could select the most appropriate game for each patient, analogous to how they select tasks in conventional therapy. The developed games engaged patients in complex, multi-joint tasks that naturally involve various muscular groups and degrees. However, each game was designed to target a primary movement component, hence pointing at a specific therapeutic goal.

1. Reaching Game: Participants have to reach for virtual balls appearing in front of them. The primary aim is to rehabilitate shoulder flexion and extension. The electrical stimulation is constant, with Gaussian-like pulse-width spikes when the target balls are reached.
2. Shapes Game: Participants have to sequentially catch color-coded balls positioned to form predefined shapes of triangles, hearts or stars. The shapes’ trajectories are positioned on a frontal plane located at arm’s distance from the participant. The game involves all of the shoulder degrees of freedom. Electrical stimulation is constant, with Gaussian-like pulse-width spikes when the balls are reached.
3. Goalkeeper Game: Participants have to sequentially block virtual soccer balls arriving from different angles, simulating the actions of a goalkeeper in a soccer game. The main goal is to rehabilitate shoulder adduction and abduction. The participants receive a constant, 1-second-long stimulation triggered by the contact of the impaired hand with the virtual ball.
4. Baseball Game: Participants have to grasp a virtual baseball bat to hit color-coded balls in sequence. The game focuses on rehabilitating shoulder internal and external rotation (on the transverse plane). The stimulation is constantly applied from the moment the participant grasps the virtual bat, with Gaussian-like pulse-width spikes superimposed upon hitting each ball.
5. Gardening Game: Participants have to use a watering can, using forearm pronation and supination to water virtual plants. This game primarily aims to rehabilitate forearm pronation and supination. The electrical stimulation is applied continuously from when the can is grasped until it is released.
6. Biceps Game: Participants have to perform biceps curl exercises to catch virtual balls. The primary goal is to rehabilitate elbow flexion and extension. Targeted stimulation is delivered as a constant base signal during hand interaction, with Gaussian-like pulse-width spikes upon contact with a ball.
7. Motorbike Game: Participants drive a virtual motorbike, accelerating and decelerating to position it in specific target areas. This game primarily targets wrist palmar flexion and extension. The stimulation is triggered upon grasping the motorbike’s handlebars and modulated based on the motorbike’s velocity.
8. Cleaning Game: Participants have to clean a virtual glass by moving their hand to ‘wipe’ dirty sections. The main aim is to rehabilitate wrist radial and ulnar deviation. The electrical stimulation is triggered upon contact with the glass and sustained during the cleaning motion.
9. Pinch Game: Participants have to pinch and release virtual objects into designated areas. This game focuses on rehabilitating hand opening and closure. The stimulation is triggered upon pinching the object. The pulse width modulates to its maximum following a Gaussian-like profile, stopping when the object is released.

For each game, the experimenter could select a difficulty level ranging from 0 to 1, tailored to the patient’s level of impairment. For example, in the Motorbike Game, setting a difficulty level of 0.5 instead of 1 reduced the required range of wrist palmar flexion and extension needed to accelerate the motorbike.

### Study design

The platform testing consisted of two phases: a pilot study to evaluate the platform’s usability and short-term effects on kinematics and body representation, followed by a randomized feasibility trial that serves as a proof-of-concept study to assess the platform’s clinical and rehabilitative impact.

The pilot study involved a single-day protocol where patients completed tasks under two experimental conditions, MultiSensy and control, with the order randomized to minimize bias (Fig. 2d). At first, electrical stimulation was calibrated to determine optimal, individualized parameters using a custom-made Python graphical user interface (GUI). Stimulation was delivered using biphasic, rectangular, charge-balanced pulses at 50 Hz^113–115^.

In the calibration phase, participants received ramps of increasing amplitude (*A*) and fixed pulse width (*PW* = 100 μs) and were instructed to report a 5/10 intensity. Electrode placement and stimulation amplitude were adjusted until the sensation was somatotopic, spreading along the median nerve across the palm and fingers. Subsequently, perceptual (minimum, 2/10 intensity) and maximum (below pain, 8/10 intensity) thresholds were determined using ramps of fixed *A* (the one saved) and increasing *PW*^116–118^. Participants repeated this process three times, and the mean *PW* values for the minimum and maximum thresholds were saved for modulating stimulation during rehabilitation games. After the calibration of the stimulation, patients underwent a Body Landmark Test to assess their baseline body perception (Fig. 2d). In the MultiSensy condition, patients performed two consecutive rounds of the Shapes Game within the immersive neurostimulation rehabilitation platform (see the Rehabilitation games section), each lasting 4 minutes (Fig. 2d). During these tasks, synchronous and targeted electrical stimulation of the median nerve was provided. After the tasks, patients repeated the Body Landmark Test and completed an embodiment questionnaire (Supplementary Table 4)^47,61^ and the SUS (Supplementary Table 5)^62^ questionnaire (Fig. 2d). In the control condition, patients performed the same Shapes Game movements without VR or electrical stimulation. Instead, posters of predefined dimensions, matching the virtual shapes, were placed on a wall (Fig. 2d). Patients faced the wall and followed the shapes with their impaired hand for two 4-minute sessions. After completing the control task, patients repeated the Body Landmark Test (Fig. 2d). While performing the movements, participants still wore VIVE trackers on their limbs, enabling the collection of kinematic data during the control condition using the same equipment as in the MultiSensy condition.

The rehabilitative impact of the platform was tested in a randomized feasibility study (Extended Data Fig. 1). Patients were randomized into one of two groups: the MultiSensy group or the conventional rehabilitation group. Randomization was conducted using a custom MATLAB script provided by an independent third party to ensure allocation concealment. The required sample size was calculated using G*Power, based on an effect size of 1.38 for immersive VR rehabilitation compared to conventional rehabilitation in improving the FMA-UE^28^ score in stroke survivors^42^. The parameters of *α* = 0.05 and statistical power of 0.90 resulted in a minimum of 12 participants per group. Both groups participated in 3 weeks of dose-matched upper-limb rehabilitation, consisting of 12 1-hour sessions. The original inclusion criterion required an FMA-UE score between 16 and 60. During enrollment, an eligibility-related protocol deviation occurred when clinicians deemed a participant with a score below this threshold suitable for inclusion. The clinicians’ rationale was reviewed by the hospital ethics committee, after which the FMA-UE lower inclusion limit was broadened to 10. In addition, a post hoc sensitivity analysis confirmed that inclusion of these participants did not influence the overall results, thereby supporting the appropriateness of including more severely impaired individuals. In the MultiSensy group, participants completed their rehabilitation within the immersive neurostimulation platform, performing 6–8 different rehabilitation games per session, each lasting a minimum of 4 minutes (Extended Data Fig. 1). Games and difficulty level selection were conducted in collaboration with physiotherapists and tailored to each participant based on their baseline FMA-UE score, ensuring the targeted rehabilitation of specific muscular components (Supplementary Table 11). Before the start of each session, participants underwent a calibration procedure as previously described. For each participant, the electrode locations and the electrical stimulation parameters were tailored to optimize somatotopic accuracy of the referred sensation. On the first day, this preparation and calibration phase required approximately 5–10 minutes; on subsequent sessions, it was reduced to 2–5 minutes, as electrode positions and stimulation settings were fine-tuned based on the saved parameters from the previous session. Participants’ stimulation parameters and location of the reported sensation can be found in Supplementary Table 12 and Supplementary Fig. 2.

In the conventional rehabilitation group, participants underwent therapist-guided upper-limb rehabilitation sessions, matched in duration to those provided by the VR platform (Extended Data Fig. 1). Upper-limb conventional rehabilitation combined physiotherapy and occupational therapy. Physiotherapy focused on maintaining muscle length, reducing spasticity if present and facilitating movement across the three-dimensional space of the impaired arm. It also included exercises to improve motor control, coordination and proprioception of the affected arm. Occupational therapy targeted functional arm activities and daily living tasks. Both physiotherapy and occupational therapy were individually tailored to each participant based on their level of impairment. Only on the first and last session days, participants in the conventional rehabilitation group performed a single Shapes Game (4 minutes) to collect kinematic movement indices at the start and end of the rehabilitation period (see the ‘Machine-learning-powered kinematic analysis of movement’ section).

The assessment timepoints and outcome measures were identical for both groups. Participants underwent assessments at four timepoints: T0 (baseline, before the intervention, day 1), T1 (mid-intervention, after six rehabilitation sessions, day 10), T2 (end of the intervention, day 19) and T3 (follow-up post-intervention, 2 weeks after T2, day 33) (Extended Data Fig. 1). At each assessment, clinical, body perception and sensory measures were collected. Primary outcomes included upper-limb motor and functional recovery, assessed using the FMA-UE^28^ and the ARAT^119^, respectively, and self-body representation, assessed using a VR-based adaptation of the Body Landmark Test^60^. For the secondary outcomes, the Barthel Index^120^ was used to evaluate the level of assistance required for ADLs; sensory recovery was assessed through tactile acuity using the 2PD test^18,99^; and PPS was assessed with a visuo-tactile PPS task^112^.

### Clinical motor assessment

All clinical assessments were conducted by therapists blinded to participants’ group allocation. The FMA-UE^28^ assesses impairments in the upper extremity, wrist, hand and coordination across 33 items. Items are scored on a 3-point scale (0 = cannot perform, 1 = performs partially, 2 = performs fully), with a total score ranging from 0 to 66, reflecting the degree of motor impairment. The ARAT^119^ evaluates upper-extremity functional performance through 19 items divided into four subtests: grasp, grip, pinch and gross arm movement. Each item is rated on a 4-point ordinal scale: 3 = performs normally, 2 = completes with difficulty, 1 = performs partially, 0 = cannot perform. To assess the degree of assistance required for ADLs, the Barthel Index^120^ was used. It measures the time and physical assistance needed for specific ADLs with a total score ranging from 0 to 100, where higher scores indicate greater independence. Each of the 10 ADL items is rated as 0 = unable, 5 = needs help (verbal, physical or assistive aid) or 10 = independent.

### Body representation assessment

**Extended Data Fig. 2:**
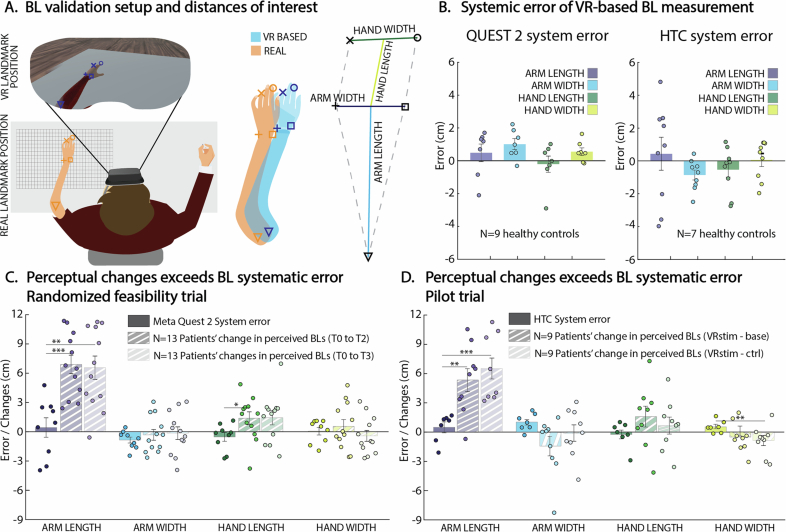
Body Landmark validation. **A**) BL validation setup and distances of interest. Baseline validation setup and distances of interest. During VR immersion, healthy controls placed their hand on a table. The real-world positions of five landmarks were recorded using a millimeter grid beneath the arm, while VR landmark positions were measured within the virtual environment. Arm and hand dimensions were assessed in both real (pink) and VR-based (blue) conditions. **B**) BL indexes systematics errors. Meta Quest 2and HTC Vive Pro errors in computing the indexes of interest are reported. The difference between VR-based and real indexes is shown for N = 9 healthy controls (Meta Quest 2) and N = 7 (HTC Vive Pro). Mean and SEM are presented. **C**) BL results from the proof-of-concept study - validation. For each index, the Meta Quest 2 system error is reported alongside the improvement from T0 to T2 and T3 for N = 13 patients. Mean and SEM are shown. Two-sided independent t-tests were used to assess whether the observed improvements were significantly greater than the system error. **D**) BL results from pilot phase - validation. For each index, the HTC system error is reported alongside the improvement from the baseline and control condition to the VRstim condition N = 9 patients. Mean and SEM are shown. Two-sided independent t-tests were used to assess whether the observed improvements were significantly greater than the system error.

Body representation was tested via a VR-based adaptation of the Body Landmark Test^60^ and a visuo-tactile PPS task^112^. The Body Landmark Test assesses short-term body representation by encoding the current position and angles of the upper limb^19^. In the original version^60^, participants sat with their forearm fixed on a table, hidden from view by a wooden frame, and verbally guided the experimenter to move a marker to align with the perceived position of five anatomical landmarks: index finger, annular finger, radius styloid, ulnar styloid and olecranon. Discrepancies between the actual (recorded while blindfolded) and perceived positions of these landmarks were measured. In our VR-based setup, participants positioned their forearm on a table with a virtual black panel displayed above the arm. They verbally guided the experimenter to move a laser pointer over the virtual panel to align with the perceived positions of the landmarks. For each participant, this was repeated five times for each of the landmarks (25 repetitions in total, randomized), and the average position of each landmark was extracted. From these data, four indices were calculated for both actual and perceived landmark positions: Arm Length (olecranon to the midpoint of radius styloid and ulnar styloid), Arm Width (distance between radius styloid and ulnar styloid), Hand Length (midpoint of radius styloid and ulnar styloid to the midpoint of index finger and annular finger) and Hand Width (distance between index finger and annular finger). Ratios of perceived to actual dimensions for each index were extracted, with a ratio of 1 indicating a perfect match. The validity of the VR-based Body Landmark Test system was assessed in a validation study with 16 healthy controls using both the Meta Quest 2 and HTC VIVE Pro headsets. The study revealed minor systematic errors (<2 cm), which were significantly lower than the improvements observed during the trial for the indexes of interest (Extended Data Fig. 2).

PPS could be defined as ‘the space immediately surrounding our bodies’^121^, ‘where human–environment interactions take place through multisensory integration’^20^. It is well established that reaction times to stimuli (for example, vision and touch) become faster when these stimuli occur within the boundaries of the PPS (that is, closer to the body). The virtual PPS testing scenario involved participants seated at a table with their impaired hand positioned on a VR-based marker 0.5 m from their head. A virtual ball appeared 1.5 m away from the marker and moved toward it at a speed of 0.75 m s^−1^. During trials, 100-ms electrical pulses were randomly delivered to the impaired hand at five fixed distances (0.93 m, 0.71 m, 0.5 m, 0.27 m and 0 m from the marker). Participants were instructed to press a trigger on the VR-based controller (using the unimpaired hand) as soon as they felt the stimulation. Each distance was tested across 15 trials (75 experimental trials in total), along with 25 baseline trials (electrical stimulation only, no ball) and 15 catch trials (no stimulation despite the looming ball). Reaction times to the electrical stimulation were recorded for all trials.

### Sensory assessment

To assess sensory recovery after the intervention, tactile acuity was assessed with the 2PD test^122^. Participants were touched on the palm of the hand with either one or two pins at a fixed distance and asked to identify the number of pins. At T0, the pin distance was individually calibrated by repeatedly increasing the pin distance from 2 mm, with 2-mm increments. For each distance, participants were touched six times with both pins and asked to identify whether they felt one or two pins. The smallest distance at which participants correctly identified at least three out of six stimuli was selected, along with the next larger distance (2 mm longer). At these two distances, participants were touched 20 times each (*n* = 40) with either one or two pins, and the percentage of correct responses was recorded as the outcome measure. At subsequent assessment timepoints (T1, T2 and T3), the exact same distances calibrated at T0 were used for testing.

### Machine-learning-powered kinematic analysis of movement

While participants performed rehabilitation tasks within the immersive neurostimulation platform, the system seamlessly collected hand and arm pose data (positions and angles) at a fixed rate of 50 Hz. These data were used to extract kinematic indices of movement for each game, providing an objective assessment of motor recovery across sessions. Kinematic indicators were extracted and grouped accordingly to ref. ^59^ in efficiency, efficacy, accuracy, smoothness and speed indicators (Supplementary Table 10). Of these, the indicators from the Shapes and Baseball games were analyzed using machine learning techniques. Missing data for specific days were handled using Iterative Imputer (max_iter = 10). First, PCA was performed to determine the number of components needed to explain the variance in the data. The correlation between PC1 and clinical scales, specifically FMA-UE and ARAT, was computed to evaluate whether the kinematic indices were linked to patients’ clinical status. Next, the principal components explaining up to 90% of the variance were used in an unsupervised *k*-means clustering analysis to test whether participants could be stratified into clinically meaningful levels of disability using kinematic variables alone. In this analysis, each data point corresponded to a single kinematic observation from one patient during one therapy session. Because FMA-UE and ARAT were assessed at only three timepoints (T0, before session 1; T1, after session 6; and T2, after session 12), intermediate values were linearly interpolated between these assessments to assign each therapy session a corresponding clinical status. Finally, we tested whether the resulting kinematic clusters corresponded to significantly different levels of motor impairment, as quantified by FMA-UE and ARAT scores.

### Statistical analysis

For all analyses, parametric or nonparametric tests were selected based on data normality, as assessed by the Kolmogorov−Smirnov test. Clinical outcomes, including improvements in FMA-UE, ARAT and Barthel Index, were initially evaluated against predefined thresholds for clinically meaningful improvements rather than statistical tests. Clinical meaningful thresholds were set at 5.25 points for FMUE^65^, at 5.7 points for ARAT^29^ and at 9.25 points for the Barthel Index^123^. Group and time effects on FMA-UE, ARAT and Barthel Index were analyzed using a linear mixed-effects model with fixed effects of Group (MultiSensy versus Conventional), Time (T0–T3) and their interaction and random intercepts for participants. The reference levels were Group = Conventional and Time = T0. Post hoc comparisons of baseline-corrected change scores (for example, ΔFMA-UE) were performed using either parametric *t*-tests or nonparametric Mann−Whitney tests, depending on data normality, to evaluate between-group differences at each timepoint. Additionally, a secondary analysis was conducted to assess whether treatment efficacy varied with participant chronicity. An ANCOVA was fitted, including FMA-UE, treatment group, months since stroke and their interactions as parameters. Detailed results are provided in the supplementary materials.

Similarly, between-group comparisons for 2PD improvements were conducted using the same statistical approach. Body Landmark Test indices were analyzed using repeated-measures ANOVA (or Friedman tests, depending on normality) to evaluate variations between assessment points among groups. Least significant difference (LSD) post hoc tests were applied for multiple comparisons where appropriate. For PPS changes analysis among groups, a two-factor (5 distances × 4 assessment timepoints) within-subjects ANOVA was performed to identify significant interaction effects, indicating multisensory facilitation (reduction in reaction times) occurring at different distances depending on the timepoints. In cases where a significant interaction was observed, PPS was calculated for each timepoint as the distance at which reaction times became significantly faster than baseline (paired *t*-test or Wilcoxon test, depending on normality). For the kinematic analysis, single indicators were compared across days within the MultiSensy group using repeated-measures ANOVA (or Friedman test, depending on normality), followed by LSD post hoc tests. In the conventional rehabilitation group, paired *t*-tests (or Wilcoxon tests, depending on normality) were used to compare intervention 1 and intervention 12. Between-group improvement analyses were conducted using independent *t*-tests (or Mann−Whitney tests, depending on normality). Correlations between kinematic principal components and FMA-UE/ARAT scores were analyzed using Pearson’s correlation. *k*-means identified clusters were compared using ANOVA, followed by LSD post hoc tests for multiple comparisons. For each analysis, all available valid observations were used for descriptive summaries. For statistical tests, missing data were handled according to the respective model used. Data were analyzed using custom scripts written in MATLAB (R2024a; MathWorks) and Python (version 3.13).

### Reporting summary

Further information on research design is available in the Nature Portfolio Reporting Summary linked to this article.

## Online content

Any methods, additional references, Nature Portfolio reporting summaries, source data, extended data, supplementary information, acknowledgements, peer review information; details of author contributions and competing interests; and statements of data and code availability are available at 10.1038/s41591-026-04486-4.

## Supplementary information

Supplementary Information Supplementary Results, Figs. 1–3, Tables 1–12 and study protocol.

Reporting Summary

Supplementary Video 1 Patient P03.

Supplementary Video 2 Patient P07.

## Data Availability

The data that support the findings of this study are available on Zenodo (10.5281/zenodo.16043228 (ref. ^124^)). The study protocol is included in the Supplementary Information.

## Extended data

**Extended Data Table 1:** Inclusion and exclusion criteria – pilot and randomized feasibility study

Pilot study		Randomized feasibility study	
Inclusion criteria	Exclusion criteria	Inslusion criteria	Exclusion criteria
Ischemic or hemorrhagic stroke patient	Prior neurological or psychiatric disorders	Ischemic or haemorrhagic stroke patient	Prior neurological or psychiatric disorders
	Severe cognitive impairment (MoCA score <10)	At least 3 months after the stroke incident	Severe cognitive impairment (MoCA score <10)
	Epilepsy	10 <= FMUE <= 60 (motor function)	Epilepsy
	Pacemakers or other electronic implants		Pacemakers or other electronic implants
	Unable to give an informed consent form		Unable to give an informed consent form

The first two columns list the inclusion and exclusion criteria for the pilot study, while the third and fourth columns list those for the randomized feasibility study.

**Extended Data Table 2:** Pilot phase participants’ (PP) characteristics

	Age (years)	Sex	Months Since Stroke	Impaired arm	FMUE	ARAT	Kinematic baseline data (mean velocity m/s)
**PP01**	71	F	85	Left	45	46	0.2552
**PP02**	53	M	7	Left	36	29	0.1226
**PP03**	48	M	2	Right	52	54	0.2410
**PP04**	53	M	112	Right	56	57	0.2377
**PP05**	68	M	3	Right	21	19	0.2490
**PP06**	63	M	20	Left	52	57	0.2397
**PP07**	62	M	10	Right	49	57	0.2846
**PP08**	60	M	1	Left	57	57	0.3135
**PP09**	55	M	85	Left	53	55	0.1837

**Extended Data Table 3:** Randomized feasibility study participants’ clinical data

	ARAT T0	ARAT T1	ARAT T2	ARAT T3	FMUE T0	FMUE T1	FMUE T2	FMUE T3	BI T0	BI T1	BI T2	BI T3
**P01**	37	48	50	55	44	44	51	54	85	90	90	90
**P02**	40	45	48	47	45	52	55	59	95	95	95	95
**P03**	28	40	45	45	41	52	58	58	95	95	95	95
**P04**	32	33	53	54	33	38	43	43	70	75	75	90
**P05**	40	42	47	46	40	44	46	57	90	90	90	90
**P06**	55	55	56	56	51	51	58	62	95	95	95	95
**P07**	0	15	15	22	13	16	32	36	75	85	85	85
**P08**	3	9	9	9	21	28	29	36	90	90	95	95
**P09**	52	52	55	55	51	57	58	61	90	90	95	95
**P10**	3	5	5	5	23	26	30	30	80	85	90	90
**P11**	31	37	37	37	35	44	45	50	80	80	80	80
**P12**	53	53	53	53	42	42	47	51	85	90	95	95
**PC1**	38	43	46	44	43	49	57	57	90	90	90	90
**PC2**	57	57	57	57	53	61	64	64	90	95	95	95
**PC3**	0	0	0	0	16	20	21	23	80	80	80	80
**PC4**	29	33	29	29	50	49	49	49	95	95	95	95
**PC5**	57	57	57	57	60	61	64	64	85	95	95	95
**PC6**	57	57	57	57	56	56	60	62	95	95	95	95
**PC7**	20	24	24	25	29	32	37	39	75	75	80	80
**PC8**	0	0	3	3	15	16	19	22	95	95	95	95
**PC9**	36	40	43	43	34	38	41	51	90	90	90	90
**PC10**	3	3	3	4	10	10	12	12	65	65	75	90
**PC11**	21	-	21	21	49	-	52	52	55	-	55	55
**PC12**	43	-	43	49	43	-	56	57	95	-	95	95
**PC13**	57	-	57	57	53	-	57	57	85	-	85	85

ARAT, FMUE, and Barthel Index for all participants across assessment points.

**Extended Data Table 4:** Randomized feasibility study Body Landmark results for Multisensy group

	RatioLengthHand	RatioWidthHand	RatioLengthArm	RatioWidthArm	GIHand	GIArm
**T0 mean±SEM**	0.831 ± 0.042	1.380 ± 0.150	0.709 ± 0.039	1.501 ± 0.112	1.738 ± 0.198	2.145 ± 0.152
**T1 mean±SEM**	0.806 ± 0.045	1.533 ± 0.166	0.916 ± 0.028	1.626 ± 0.100	1.998 ± 0.209	1.785 ± 0.111
**T2 mean±SEM**	0.890 ± 0.028	1.434 ± 0.111	0.979 ± 0.021	1.325 ± 0.100	1.658 ± 0.123	1.376 ± 0.116
**T3 mean±SEM**	0.890 ± 0.011	1.202 ± 0.090	0.965 ± 0.027	1.352 ± 0.045	1.351 ± 0.102	1.426 ± 0.054
**TestUsed**	RANOVA	RANOVA	RANOVA	RANOVA	RANOVA	RANOVA
**PValue**	0.026060346	0.213123656	9.78928E-09	0.005372455	0.064014321	2.42691E-06
**T0_vs_T1**	p=0.1806, d=−0.25	-	p=0.0000, d=2.00	p=0.1790, d=0.34	-	p=0.0467, d=−0.75
**T0_vs_T2**	p=0.1396, d=0.55	-	p=0.0001, d=2.06	p=0.0899, d=−0.45	-	p=0.0002, d=−1.54
**T0_vs_T3**	p=0.1836, d=0.43	-	p=0.0006, d=1.50	p=0.1853, d=−0.43	-	p=0.0013, d=−1.33
**T1_vs_T2**	p=0.0167, d=0.79	-	p=0.008, d=0.92	p=0.0034, d=−0.75	-	p=0.0017, d=−0.93
**T1_vs_T3**	p=0.0539, d=0.66	-	p=0.2136, d=0.40	p=0.0035, d=−1.14	-	p=0.0026, d=−1.20
**T2_vs_T3**	p=0.8576, d=0.06	-	p=0.3419, d=−0.30	p=0.7024, d=0.12	-	p=0.5827, d=0.17

Body Landmark indexes from N = 12 participants are presented. Mean ± SEM values are reported for T0, T1, T2, T3 and along with the statistical test used, corresponding p-value, and Cohen’s paired effect size.

**Extended Data Table 5:** Randomized feasibility study Body Landmark results for conventional rehabilitation group

	RatioLengthHand	RatioWidthHand	RatioLengthArm	RatioWidthArm	GIHand	GIArm
**T0 mean±SEM**	0.958 ± 0.047	1.675 ± 0.117	0.700 ± 0.031	1.915 ± 0.114	1.821 ± 0.169	2.812 ± 0.186
**T1 mean±SEM**	0.821 ± 0.028	1.537 ± 0.147	0.663 ± 0.029	1.878 ± 0.134	1.888 ± 0.161	2.880 ± 0.262
**T2 mean±SEM**	0.838 ± 0.033	1.650 ± 0.132	0.672 ± 0.035	1.719 ± 0.174	2.015 ± 0.176	2.700 ± 0.352
**T3 mean±SEM**	0.867 ± 0.040	1.638 ± 0.176	0.731 ± 0.031	1.991 ± 0.138	1.953 ± 0.214	2.810 ± 0.276
**TestUsed**	RANOVA	RANOVA	RANOVA	RANOVA	RANOVA	RANOVA
**PValue**	0.002693315	0.69551277	0.137984624	0.919857313	0.856612788	0.925779955
**T0_vs_T1**	p=0.0065, d=−1.39	-	-	-	-	-
**T0_vs_T2**	p=0.0199, d=−0.73	-	-	-	-	-
**T0_vs_T3**	p=0.0408, d=−0.88	-	-	-	-	-
**T1_vs_T2**	p=0.9354, d=−0.04	-	-	-	-	-
**T1_vs_T3**	p=0.3595, d=0.35	-	-	-	-	-
**T2_vs_T3**	p=0.4496, d=0.28	-	-	-	-	-

Body Landmark indexes from N = 12 participants are presented. Mean ± SEM values are reported for T0, T1, T2, T3 and along with the statistical test used, corresponding p-value, and Cohen’s paired effect size.

## Code availability

The custom code used to process and analyze the data and generate the figures is available on Zenodo (10.5281/zenodo.16043228 (ref. ^124^)).
