# Supplementary material for: Immersive virtual reality with synchronous neurostimulation for upper-limb recovery after stroke: a randomized feasibility trial
Source: Nat Med. 2026 Jun 26;32(8):2790–802. doi: 10.1038/s41591-026-04486-4 (PMC13472961; doi:10.1038/s41591-026-04486-4)
Supplement: Supplementary file 1 [file 41591_2026_4486_MOESM1_ESM.pdf]

nature medicine

Supplementary information https://doi.org/10.1038/s41591-026-04486-4

Immersive virtual reality with synchronous neurostimulation for upper-limb recovery after stroke: a randomized feasibility trial

In the format provided by the authors and unedited

Supplementary Information

Content table:

Supplementary results

Figure S1. PPS analysis.

Figure S2. Stimulation perceived location

Figure S3. Randomized feasibility study SUS scores

Table S1. Detailed sample sizes per outcome for the randomized feasibility study

Table S2. Randomized feasibility study – participants comorbidities

Table S3. Grouped baseline analysis.

Table S4. Embodiment questionnaire.

Table S5. SUS questionnaire.

Table S6. Pilot phase kinematic analysis.

Table S7. Pilot phase Body Landmark analysis.

Table S8. Randomized feasibility study participants' clinical improvement.

Table S19. Randomized feasibility study participants' 2PD improvement.

Table S10. Kinematic features.

Table S11. Patient Game Selection.

Table S12. Stimulation parameters.

Study protocol

Supplementary results

We performed a secondary analysis to examine whether treatment efficacy varied with chronicity. Specifically, we fit an ANCOVA including baseline FMUE, treatment group, months since stroke (centered), and their interaction.

$$FMUE_{T3} = \beta_0 + \beta_1 Group + \beta_2 FMUE_{T0} + \beta_3 Months_c + \beta_4 (Group \times Months_c)$$

**Outcome / Model**	**n**
FMUE_T3 ~ Group + FMUE_T0 + Months_c + Group:Months_c	25

Control was defined as the reference level for *Group* and *Months_c = Months – mean(Months)*. All participants were included in the analysis (n = 25).

We report the model fit metrics in the following table:

**Model fit**	**Value**
R^2	0.897
Adjusted R^2	0.876
RMSE	5.09
DF error	20
F vs const	43.3
F p-value	1.4E-09

And the parameter estimates:

**Coefficient**	**Estimate**	**SE**	**t**	**p**
(Intercept)	9.2996	3.3679	2.7613	0.012044
FMUE_T0	0.95491	0.077575	12.309	8.6588e-11
Group_MS	5.4885	2.0493	2.6783	0. 014448
Months_c	0.0094714	0.018716	0.50607	0.61834
Group_MS:Months_c	-0.03115	0.030768	-1.0124	0.32343

At the mean chronicity, the adjusted group difference favored the intervention (MultiSensy vs Control = +5.49 points, SE 2.05, $p = 0.014$). Months since stroke was not a prognostic predictor after adjustment ($\beta = 0.009 \pm 0.019$, $p = 0.62$), and the Group × Months interaction was non-significant ($\beta = -0.031 \pm 0.031$, $p = 0.32$), indicating that treatment efficacy did not depend on chronicity.

Estimated treatment effects were similar at 3, 6, and 12 months post-stroke (+6.69, +6.60, and +6.41 FMUE points; all $p \approx 0.01$) as detailed in the following table:

**Simple effects (MS–C)**	**Estimate**	**SE**	**p**
3 months	6.69	2.4	0.0112
6 months	6.6	2.35	0.0108
12 months	6.41	2.27	0.0103

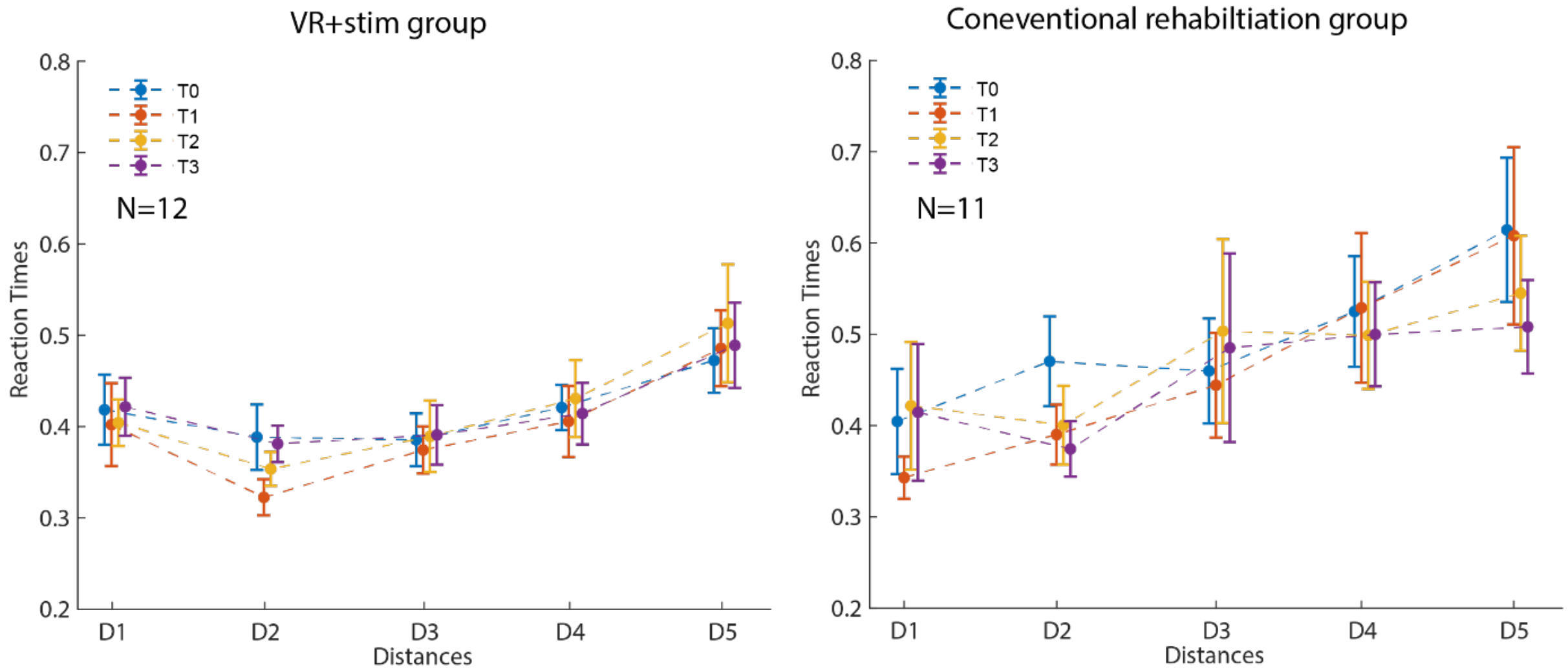

Figure S1. PPS analysis. Reaction times at different distances are shown for the VR+stim group (N=12, left) and the Conventional Rehabilitation group (N=11, right) across the four timepoints. Mean ± SEM is displayed for each distance.

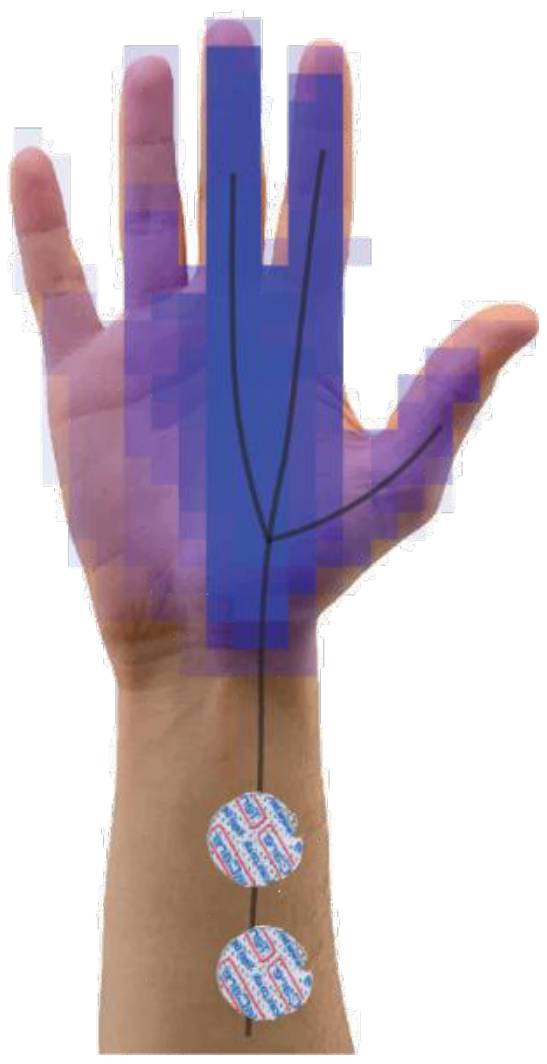

Figure S2. Stimulation perceived location. Distribution of reported sensation areas during the calibration procedure in the randomized feasibility study patients. Darker colors indicate a higher number of participants experiencing somatotopic sensations in that region.

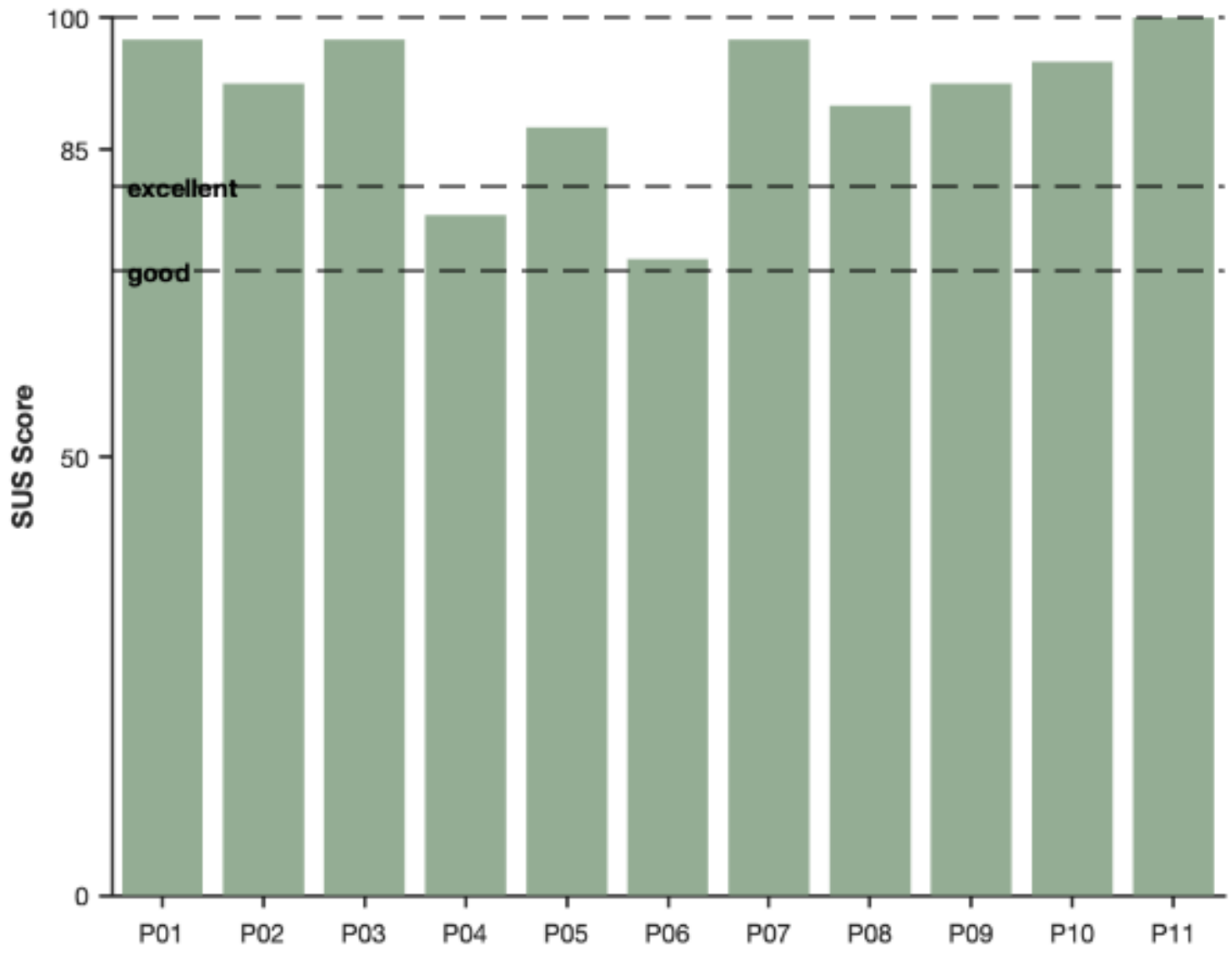

Figure S3. Randomized feasibility study SUS scores. Individual System Usability Scale (SUS) scores are shown, with horizontal lines indicating thresholds for good (71.1) and excellent (80.8) usability ratings.

Outcome	Group	T0	T1	T2	T3
**FMA-UE/ARAT/BI**	Intervention (n=12)	12	12	12	12
**FMA-UE/ARAT/BI**	Control (n=13)	13	10	13	13
**2PD**	Intervention (n=12)	12	12	12	12
**2PD**	Control (n=13)	12	9	12	9
**Body Landmark**	Intervention (n=12)	12	12	12	11
**Body Landmark**	Control (n=13)	12	9	12	8
**Kinematic measures**	Intervention (n=12)	12	N.A.	12	N.A.
**Kinematic measures**	Control (n=13)	9	N.A.	9	N.A.

Table S1. Detailed sample sizes per outcome for the randomized feasibility study

	Comorbidities
**P01**	Hypertension, Benign prostatic hyperplasia, Shoulder tendinopathy & bursitis, Bilateral knee osteoarthritis, Insomnia
**P02**	Hypertension, Generalized anxiety disorder, Peroneal nerve lesion (foot drop), Post-stroke neuropathic pain
**P03**	Hypertension, arteriovenous malformation, allergy to dairy and hazelnuts
**P04**	Thrombophilia, myocardial scar after inferior infarction, mixed hyperlipidemia, hypertension, allergy to penicillin
**P05**	Hypertension, diabetes mellitus type II, cervical spondylosis
**P06**	Colon cancer (malignant neoplasm of hepatic flexure), ileostomy, fatty liver, post-traumatic amputation of right fifth toe and left third toe
**P07**	Mitral valve replacement with mechanical valve, atrial septal defect correction, ischemic heart disease, angina pectoris, hypertension, mixed hyperlipidemia, Hashimoto's thyroiditis, hypothyroidism, gastroesophageal reflux disease
**P08**	Thyroid nodule (struma multinodosa), gastroesophageal reflux disease, dysphonia, dysphagia, depression
**P09**	Hypertension, mixed hyperlipidemia, gastroesophageal reflux disease, generalized anxiety disorder, seropositive rheumatoid arthritis
**P10**	Diabetes mellitus type II, hypertension, gastroesophageal reflux disease
**P11**	Hypertension, type II diabetes mellitus, hyperparathyroidism, metabolic amino acid disorder, gastroesophageal reflux disease, chronic pain syndrome
**P12**	Hypertension, atrial fibrillation, fluctuating aortic atherosclerosis
**PC01**	Anxiety disorder, mixed hyperlipidemia
**PC02**	Hypertension, hypercholesterolemia, metabolic disorder, depressive episode
**PC03**	Diabetes mellitus type II, hypertension, gastroesophageal reflux disease, generalized anxiety disorder
**PC04**	Diabetes mellitus, hypertension
**PC05**	Hypertension
**PC06**	Hypertension, osteoporosis, left peroneal nerve lesion, progressive vascular leukoencephalopathy
**PC07**	Hypertension, type II diabetes mellitus, hyperlipidemia, vitamin B12 deficiency, oral candidiasis, depressive episode
**PC08**	Renal osteodystrophy, cataract, hypertension
**PC09**	Hyperlipidemia
**PC10**	Hypertension, gastroesophageal reflux disease, chronic sinusitis, allergies to food and medications
**PC11**	Hypertension, type II diabetes mellitus, gastroesophageal reflux disease, carotid artery stenosis, history of bladder surgery
**PC12**	Hypertension, chronic obstructive pulmonary disease, hyperlipidemia, cardiomyopathy, ischemic heart disease, vertebral and subclavian artery stenosis, osteoporosis, allergy to ciprofloxacin and pollen
**PC13**	Hypertension, type II diabetes mellitus, diabetic polyneuropathy

Table S2. Randomized feasibility study – participants comorbidities

	VR+stim (mean +- std)	Conventional (mean +- std)	pvalue
**Age**	57.2 +- 9.4	61.6 +- 11.9	0.31
**Months since stroke**	40.4 +- 64.8	42.9 +- 84.7	0.27
**FMUE**	36.6 +- 12.1	39.3 +- 16.9	0.65
**ARAT**	31.2 +- 19.6	32.2 +- 22.1	0.91

Table S3. Grouped baseline analysis. Age, Months since stroke, and baseline FMUE and ARAT statistical analysis across groups.

**Embodiment questionnaire**	
**Component**	**Question**
**Ownership**	Q1. I had the feeling that I was looking at my body
	Q2. I felt as if the virtual body was my body
**Location**	Q4. I felt as if my body was located where I saw the virtual body
	Q5. I felt as if my (real) body was drifting towards the virtual body
**Touch**	Q6. It seemed as if I felt the stimulation in the location where I saw the virtual body stimulated
	Q7. It seemed as if the tactile sensation I felt was caused by the visual stimulus on the virtual body
**Agency**	Q9. It felt like I could control the virtual body as if it was my own body
	Q10. The movements of the virtual body were caused by my movements
**Control**	Q3. It seemed as if I might have more than one body
	Q8. It seemed as if the stimulation I felt was located somewhere between my physical body and the virtual body
	Q11. I felt as if the virtual body was moving by itself

Table S4. Embodiment questionnaire. Each question was rated on a scale from -3 to 3, where -3 corresponds to "strongly disagree," -2 to "disagree," -1 to "somewhat disagree," 0 to "neither agree nor disagree," 1 to "somewhat agree," 2 to "agree," and 3 to "strongly agree." The Embodiment Score was calculated as the mean of responses to questions Q1, Q2, Q4, Q5, Q6, Q7, Q9, and Q10, while the Control Score was calculated as the mean of responses to questions Q3, Q8, and Q11.

		Strongly Disagree	Somewhat Disagree	Neutral	Somewhat Agree	Strongly Agree
**1.**	I think I would like to use this tool frequently.	☐	☐	☐	☐	☐
**2.**	I found the tool unnecessarily complex.	☐	☐	☐	☐	☐
**3.**	I thought the tool was easy to use.	☐	☐	☐	☐	☐
**4.**	I think that I would need the support of a technical person to be able to use this system.	☐	☐	☐	☐	☐
**5.**	I found the various functions in this tool were well integrated.	☐	☐	☐	☐	☐
**6.**	I thought there was too much inconsistency in this tool.	☐	☐	☐	☐	☐
**7.**	I would imagine that most people would learn to use this tool very quickly.	☐	☐	☐	☐	☐
**8.**	I found the tool very cumbersome to use.	☐	☐	☐	☐	☐
**9.**	I felt very confident using the tool.	☐	☐	☐	☐	☐
**10.**	I needed to learn a lot of things before I could get going with this tool.	☐	☐	☐	☐	☐

Table S5. SUS questionnaire. SUS score was calculated as The SUS score was calculated as 2.5 × (sum of odd responses – 1 + 5 – sum of even responses).

Index Name	VR+stim (mean ± SEM)	control (mean ± SEM)	p-value	Statistical test	Cohen's paired d
**velocity**	0.236 ± 0.018	0.172 ± 0.028	0.025	t-test paired	0.917
**jerk**	57.552 ± 25.969	84.832 ± 34.903	0.1267	t-test paired	-0.568
**smoothness**	-3.019 ± 0.206	-3.11 ± 0.191	0.4301	t-test paired	0.277
**Frechetdistance**	0.118 ± 0.003	0.217 ± 0.018	**0.0007**	t-test paired	-1.79
**RMSE**	0.043 ± 0.003	0.055 ± 0.004	0.052	t-test paired	-0.76
**areaerror**	-0.374 ± 0.038	-0.505 ± 0.044	0.0342	t-test paired	0.85
**MovementTime**	6.746 ± 1.202	7.755 ± 0.929	0.1955	t-test paired	-0.471
**TimeBetweenShapes**	1.362 ± 0.107	1.513 ± 0.376	0.4961	Wilcoxon signed-rank	-0.13
**PeakVelocity**	0.507 ± 0.031	0.439 ± 0.07	0.232	t-test paired	0.431
**NoPeaksVelocity**	27.059 ± 3.379	24.417 ± 3.501	0.4089	t-test paired	0.291
**PronoSupination**	0.25 ± 0.061	0.088 ± 0.009	0.0212	t-test paired	0.953

Table S6. Pilot phase kinematic analysis. Kinematic data for all indicators from N=9 participants are presented. Mean ± SEM values are reported for both VR+stim and control conditions, along with the statistical test used, corresponding p-value, and Cohen's paired effect size. In bold are highlighted significant p-values for p < 0.01.

BL index	baseline (mean ± SEM)	VR+stim (mean ± SEM)	control (mean ± SEM)	RANOVA F	p-value	Poshoc p-value and (paired cohen's)		
						Baseline vs VR+stim	Baseline vs control	VR+stim vs control
Ratio LengthHand	0.708 ± 0.040	0.793 ± 0.053	0.752 ± 0.062	0.857	0.443	-	-	-
Ratio WidthHand	1.519 ± 0.184	1.369 ± 0.210	1.414 ± 0.169	0.505	0.63	-	-	-
Ratio LengthArm	0.571 ± 0.026	0.783 ± 0.039	0.499 ± 0.026	22.398	2.301-05	0.004 (d=-1.35)	0.0341 (d=0.851)	0.0004 (d=1.972)
Ratio WidthArm	2.043 ± 0.305	1.425 ± 0.141	1.599 ± 0.158	5.117	0.019	0.0274 (d=0.897)	0.0529 (d=0.757)	0.3303 (d=-0.345)
GIHand	2.304 ± 0.279	1.842 ± 0.311	2.116 ± 0.311	1.04	0.375	-	-	-
GIArm	3.837 ± 0.566	1.817 ± 0.145	3.429 ± 0.438	13.053	**0.0004**	0.0029 (d=1.404)	0.2905 (d=0.377)	0.0041 (d=-1.324)

Table S7. Pilot phase Body Landmark analysis. Body Landmark indexes from N=9 participants are presented. Mean ± SEM values are reported for baseline, VR+stim and control conditions, along with the statistical test used, corresponding p-value, and Cohen's paired effect size. In bold are highlighted significant p-values for p < 0.01.

	Assessment	VR+stim mean ± SEM	Conventional mean ± SEM	Statistical test	pvalue	Cohen's d
ARAT	T1	5.000 ± 1.497	2.429 ± 0.869	t-test	0.235	0.641
ARAT	T2	8.250 ± 1.958	2.444 ± 1.082	t-test	0.029	1.092
ARAT	T3	9.167 ± 2.374	3.111 ± 0.978	t-test	0.049	0.981
FMUE	T1	4.583 ± 1.041	2.600 ± 0.921	t-test	0.177	0.605
FMUE	T2	9.417 ± 1.252	6.000 ± 1.225	t-test	0.064	0.781
FMUE	T3	13.167 ± 1.302	7.538 ± 1.483	t-test	0.01	1.138
BI	T1	2.500 ± 0.973	1.500 ± 1.067	t-test	0.376	0.296
BI	T2	4.167 ± 1.205	2.308 ± 1.076	t-test	0.225	0.461
BI	T3	5.417 ± 1.790	3.462 ± 1.992	t-test	0.216	0.291

Table S8. Randomized feasibility study participants' clinical improvement. ARAT, FMUE, and Barthel Index improvement. Mean ± SEM improvements are reported for T1, T2, T3, along with the post-hoc statistical test used, corresponding p-value, and Cohen's paired effect size.

Assessment	VR+stim (mean ± SEM)	control (mean ± SEM)	p-value	Statistical test	Cohen's paired d
T1	0.115 ± 0.041	-0.007 ± 0.051	t-test	0.073	0.83
T2	0.162 ± 0.044	-0.006 ± 0.038	t-test	0.008	1.189
T3	0.208 ± 0.046	0.030 ± 0.028	t-test	0.006	1.41

Table S9. Randomized feasibility study participants' 2PD improvement. 2PD improvement. Mean ± SEM improvements are reported for T1, T2, T3, along with the statistical test used, corresponding p-value, and Cohen's paired effect size

	Accuracy	Efficacy	Efficiency	Smoothness	Speed
Shapes (Frontal task)	RMSE, area error, Elbow Pronosup	Score, Hit Score	Task Time, Inter-movements Time, Score Rate, Hit Rate	jerk, SPARC, NoPeaksVelocity	Velocity, PeakVelocity
Baseball (Planar task)	RMSE, area error	Score, Hit Score	Task Time, Inter-movements Time, Score Rate, Hit Rate	jerk, SPARC, NoPeaksVelocity	Velocity, PeakVelocity
Gardening	UlnarRadialROM, UlnarRadialSTD, UlnarRadialW	Score	Task Time, Score Rate	jerk, SPARC, NoPeaksVelocity	Velocity, PeakVelocity
Motorbike	WristFlexionROM, WristFlexionSTD, WristFlexionW	Score	Score		
Cleaning	UlnarRadialROM, UlnarRadialSTD, UlnarRadialW	Score	Task Time, Score Rate	jerk, SPARC, NoPeaksVelocity	Velocity, PeakVelocity
Pinch	RMSE	Score	Task Time, Score Rate	jerk, SPARC, NoPeaksVelocity	Velocity, PeakVelocity
Biceps Curls	RMSE, area error, Elbow Pronosup	Score, Hit Score	Task Time, Inter-movements Time, Score Rate, Hit Rate	jerk, SPARC, NoPeaksVelocity	Velocity, PeakVelocity
Football	AdductionROM, AdductionSTD, AdductionW,	Score	Task Time, Score Rate	jerk, SPARC, NoPeaksVelocity	Velocity, PeakVelocity
Reaching	RMSE	Score	Task Time, Score Rate	jerk, SPARC, NoPeaksVelocity	Velocity, PeakVelocity

Table S10. Kinematic features. List of all the kinematic indicators extracted for each game.

	Shapes	Reaching	Football	Baseball	Biceps curls	Gardening	Motorbike	Cleaning	Pinch
**P01**	X			X		X	X	X	X
**P02**	X			X	X	X	X	X	X
**P03**	X		X	X		X	X	X	X
**P04**	X		X	X	X	X	X	X	
**P05**	X			X	X	X	X	X	X
**P06**	X		X	X	X			X	X
**P07**	X	X	X					X	X
**P08**	X	X	X	X				X	
**P09**	X			X		X		X	X
**P10**	X	X	X	X					
**P11**	X	X	X	X	X				X
**P12**	X		X	X	X			X	X

Table S11. Patient Game Selection. The games selected by each patient from the Neuro-Platform library are listed

	Amplitude (mA) mean ± STD	Min PW (μs) mean ± STD	Max PW (μs) mean ± STD
**P01**	10.00 ± 0.00	109.17 ± 4.52	225.83 ± 9.73
**P02**	15.00 ± 0.00	129.17 ± 5.70	250.00 ± 12.61
**P03**	10.00 ± 0.00	95.83 ± 1.93	213.33 ± 17.42
**P04**	9.50 ± 0.26	81.67 ± 3.66	162.50 ± 9.93
**P05**	10.00 ± 0.00	100.00 ± 0.00	197.50 ± 4.29
**P06**	5.00 ± 0.00	104.17 ± 8.92	174.17 ± 11.25
**P07**	10.00 ± 0.00	85.00 ± 2.61	180.00 ± 4.26
**P08**	10.00 ± 0.00	93.33 ± 2.84	190.00 ± 5.77
**P09**	10.00 ± 0.00	57.50 ± 3.05	110.00 ± 5.22
**P10**	10.00 ± 0.00	60.83 ± 1.93	119.17 ± 2.60
**P11**	10.00 ± 0.00	50.00 ± 0.00	126.67 ± 5.12
**P12**	10.00 ± 0.00	32.50 ± 1.79	72.50 ± 2.50

Table S12. Stimulation parameters. Mean ± STD amplitude, min and max pulsewidth per patient of the VR+stim group across rehabilitation days.

CLINICAL INVESTIGATION PLAN

Comparison of the effects of a multimodal platform combining virtual reality and transcutaneous nerve stimulation with conventional therapy, on upper-limb rehabilitation in post-stroke patients

Reference number: MultiSensy 1.1
Version number: 1.1
Date: 15/02/2024

Sponsor: Institute Mihajlo Pupin, Volgina 15, 11060 Belgrade, Serbia;

- Principal Investigator: Dr Staniša Raspopović, Department of Health Sciences and Technology, ETH Zürich; Tannenstrasse 1, 8092 Zürich and External Senior Research Associate at Institute Mihajlo Pupin, Volgina 15, 11060 Belgrade, Serbia

Investigation site: Clinic for Rehabilitation "Dr. Miroslav Zotović", 11000, Belgrade, Serbia
External organizations involved in the clinical investigation:

- Department of Health Sciences and Technology, ETH Zürich; Tannenstrasse 1, 8092 Zürich

Table of contents

Persons involved 4
1 Introduction 5
1.1 Justification for the design of the clinical investigation 5
1.2 Description of the system 5
1.3 Risk-benefit assessment 6
2 Study objective and hypothesis 7
2.1 Primary objective 7
2.2 Secondary objective 10
3 Study Description 11
3.1 Study design 11
3.2 Study centre 11
3.3 Responsible ethics committee 11
3.4 Registration of the study 11
3.5 Time schedule 11
4 Methods 12
4.1 Study Population 12
4.1.1 Inclusion and exclusion criteria 12
4.1.2 Engagement of vulnerable groups 12
4.1.3 Number of participants and sample calculation 12
4.2 Intervention 13
4.2.1 Therapy scheme 13
4.2.2 Assessment of safety and side effects 16
4.2.3 Compatibility/ safety parameters 16
4.3 Adverse events 16
4.3.1 Definitions 16
4.3.2 Definition of adverse events 16
4.4 Serious adverse events (SAEs) 17
4.4.1 SAEs related to the intervention 17
4.4.2 Suspected unexpected serious adverse reactions (SUSARs) 17
4.5 Severity of adverse events 18
4.6 Association with the study intervention 18
4.7 Reporting procedures 19
4.7.1 Reporting procedures for SAEs 19
4.7.2 Reporting procedures for SUSAR 20
5 Statistical methodology 20

5.1 Source data 20
5.2 Case Report Form (CRF) 20
5.3 Data Analysis 21
5.3.1 Statistical Data Analysis 21
5.4 Storage of study documents, data storage & deletion 23
5.5 Data Management 24
5.5.1 Data input and export 24
5.5.2 Quality Ensuring Measures 24
6 ETHICAL AND LEGAL ASPECTS 24
6.1 Informed consent of subjects 24
6.2 Acknowledgement / approval of the study 25
6.2.1 Changes in the conduct of the study 25
6.3 Ethics and good clinical practice (GCP) 25
7 REFERENCES 26

Persons involved

Function	**Name & Contact**
Sponsor	**Institute Mihajlo Pupin**<br>Volgina 15, 11060<br>Belgrade, Serbia
Principal Investigator	**Prof. Stanisa Raspopovic**<br>**ETH Zurich**<br>Department of Health Sciences and Technology,<br>Tannenstrasse 1, 8092 Zürich<br>Email: stanisa.raspopovic@hest.ethz.ch
Coordinating Investigator	**Prof. MD. Ljubica M. Konstantinovic**<br>**Clinic for Rehabilitation "Dr. Miroslav Zotović"**<br>Sokobanjska 13, Beograd 11000, Serbia<br>Email: ljkonstantinovic@yahoo.com
Research Collaborator	**MD. Olivera Djordjevic**<br>**Clinic for Rehabilitation "Dr. Miroslav Zotović"**<br>Sokobanjska 13, Beograd 11000, Serbia<br>Email: odordev@gmail.com
Research Collaborator	**Giuseppe Valerio Aurucci**<br>**ETH Zurich**<br>Department of Health Sciences and Technology,<br>Tannenstrasse 1, 8092 Zürich<br>Email: giuseppevalerio.aurucci@hest.ethz.ch
Research Collaborator	**Dr. Natalija Secerovic**<br>**Institute Mihajlo Pupin**<br>Volgina 15, 11060 Belgrade, Serbia;<br>Email: natalija.katic@pupin.rs

1 Introduction

1.1 Justification for the design of the clinical investigation

Stroke is a disabling medical condition annually affecting up to 15 million people worldwide[1]. It leads to upper-limb impairments encompassing motor and sensory deficits[2] together with cognitive self-body and space misrepresentation[3], overall limiting the functional independence of 70% of stroke survivors[4]. On the motor side, stroke could account for hemiparesis (weakness or paralysis affecting the side contralateral to the brain lesion), muscle weakness, spasticity, loss of coordination, and others[5]. On the sensory side, especially in the first stages after the stroke occurs, stroke could account for sensory loss, with the patient not being able to perceive what he's touching with the impaired arm.[4] On a cognitive level, it has been shown that chronic stroke patients have distorted body representation and space representation. They perceive their impaired arm as shorter and the impaired hand as larger[3]. Despite initial evidence of the crucial role of sensory-motor integration[6,7] toward a restored body representation[8] to promote effective rehabilitation, conventional approaches suffer from the bias of prioritizing motor recovery while disregarding stroke-induced sensory and body representation deficits.
In this view, the creation of a virtual reality (VR) scenario in which the person is fully immersed could potentially play a significant role in improving stroke patients' rehabilitation.
Taking this into consideration, this project aims to assess whether a multimodal platform combining VR with TENS inducing full-body illusion toward a virtual avatar could positively impact motor performances, sensory assessments, and self-body and space representation of stroke patients.

1.2 Description of the system

Technologically the project is characterized by two main components: TENS stimulation and a system of Virtual Reality (shown in the images below).
The TENS (Transcutaneous Electrical Nerve Stimulation) (Figure 1) system is composed of the RehaMove stimulator used during the experiment to give the subject a tactile sensation similar to a vibration to his/her hands; the aim is to induce a somatotopic sensation, meaning that it is perceived in a wider area and not just below the electrodes. The current level involved are in the range of 4-12mA, but an initial phase of calibration is needed to find the level of current that best adapts to each location and each subject. The stimulation is pulse-width-modulated, meaning that it varies between a minimum and maximum pulse width (both found during calibration).

[redacted]

Figure 1: TENS device

The Virtual Reality (VR) system is the OCULUS QUEST META 2 headset (Figure 2), composed of a headset that sets the subject in an immersive virtual environment (refresh rate of 90Hz) with a built-in hand recognition system to perform real-time motion tracking.

[redacted]

Figure 2: VR device

More into detail, the intervention will consist of the patient performing some task-oriented movement within the virtual reality and congruently tactile receiving feedback through transcutaneous electrical nerve stimulation. The patients will indeed be asked to follow predefined shapes constructed with interactive elements within the 3D space. He will receive electrical feedback every time his virtual hand touches a virtual element within the shape. Also, goal-oriented tasks will be performed. Here, the subject will receive clear instruction within the virtual reality scenario to perform specific actions toward a final goal. These actions will be designed to make the subject repeat some crucial movements in his rehabilitation process. Depending on the motor impairment of the patient, we will adapt the characteristics and the difficulty of the task accordingly

1.3 Risk-benefit assessment

This study aims to examine the effects of multimodal platforms providing both VR and TENS therapies for stroke rehabilitation. Both systems we use are CE-approved, so the risks are minimal. Electrical stimulation may cause some discomfort sensation. However, we always conduct a calibration procedure before each experiment to determine the optimal electrical

stimulation parameters for each patient, and we provide constant supervision throughout the session. The stimulation will not be perceived as painful due to hardware limitations on the current levels. For VR, the primary concern may be a sensation of vertigo. However, vertigo typically results from a discrepancy between vestibular and visual system information. In our setup, this does not occur because the patient remains seated, and the first-person avatar they see in VR mirrors their real movements. This alignment minimizes any conflict between vestibular sensation and vision. Additionally, participants are free to interrupt the experiment if they suddenly feel uncomfortable. Additionally, the potential quality of life benefits that would come with the improvement of motor, sensory, and proprioceptive deficits are thought to outweigh these risks.

Benefits

The benefits of using the system come from the improved motor ability to increased functionality of stroke patients. Also, being the intervention multimodal, the subject would benefit from increased sensitivity on the paretic hand as well as improved proprioception and body representation.
Moreover, if successful, the study will be one step towards the development of a new commercially available rehabilitation method at home.

Risks

1) Discomfort sensation following electrical stimulation.
   a. *Cause:* Injection of electrical charge.
   b. *Mitigation:* Hardware limits on the injected current. The calibration step performed before the experiment to ensure the best stimulation parameter for each subject. Constant human supervision.

2) Vertigo from virtual reality therapy.
   a. *Cause:* wearing the virtual reality headset and watching a moving video.
   b. *Mitigation:* Participants are always seated during the protocol, and they are told they can choose not to perform a task if they suddenly feel uncomfortable.

2 Study objective and hypothesis

2.1 Primary objective

This project aims to investigate whether a multimodal platform combining VR with transcutaneous electrical nerve stimulation (TENS) can positively influence mobility, sensory perception, body schema, and spatial awareness in stroke patients after a 3-week-long rehabilitation protocol.

To this end, these are the main outcomes of interest:

1) FMUE [9]

- *Aim:* To assess reflex activity, movement control and muscle strength in the upper extremity of people with post-stroke hemiplegia.
- *Test Performance:* The FMUE Scale comprises 33 items, each scored on a scale of 0 to 2, where 0 = cannot perform, 1 = performs partially and 2 = performs fully.
- *Results Interpretation:* The final score is calculated by summing the value of individual scores and will classify the patient in 4 categories (Severe, Severe to Moderate, Moderate to Mild and Mild)

2) ARAT [10]

- *Aim:* To assess functional performance of the upper extremity through observational means.
- *Test Performance:* The ARAT is a 19-item measure divided into 4 sub-tests (grasp, grip, pinch, and gross arm movement). Performance on each item is rated on a 4-point ordinal scale ranging from: 3) Performs test normally 2) Completes test, but takes abnormally long or has great difficulty 1) Performs test partially 0) Can perform no part of test.
- *Results Interpretation:* The final score is calculated by summing the value of individual scores and will classify the patient.

3) Body Landmark test [3]

- *Aim:* To measure the body representation of the subjects
- *Test Performance:* In VR, the subject is asked to locate the position of five specific body landmarks (elbow, inner wrist, outer wrist, index, ring) while a black panel is on top of his/her arm.
- *Results Interpretation:* Indexes are extracted to show how much a patient is over or underestimating the dimension of arm and hand.

Other outcomes of interest are:

4) Barthel Index [11]

- *Aim:* To assess the degree of assistance required by an individual on ten mobility and self-care ADL items.
- *Test Performance:* The time taken and physical assistance required to perform each item are used to determine the assigned value of each item.
- *Results Interpretation:* The ten items are scored with a number of points, and then a final score is calculated by summing the points awarded to each functional skill. This allows the examiner to measure a patient's functional disability by quantifying their performance.

5) Two-point discrimination test [12]

- *Aim:* To measure the tactile acuity of patients
- *Test Performance:* While blindfolded, the patient is repetitively touched with either one or two pins (fixed distance) and he asked to tell how many pins he/she feels.
- *Results Interpretation:* The final score is calculated as a percentage of correctly classified samples.

6) Peri Personal Space test [3]

- *Aim:* To measure the peri-personal space of stroke patients (the space in which multisensory integration is enhanced)
- *Test Performance:* In VR, the subject is sitting on a table and sees balls approaching him. He/she's asked to press a controller whenever he/she feels electrical stimulation.
- *Results Interpretation:* The precise distance from which multisensory integration is enhanced (PPS) will correspond to the space in which the subject feels confident to act.

7) Ashworth Scale for shoulder, elbow and wrist [13]

- *Aim:* To test resistance to passive movement about a joint with varying degrees of velocity.
- *Test Performance:* It's performed by extending the patient's limb first from a position of maximal possible flexion to maximal possible extension (the point at which the first soft resistance is met). Afterwards, the modified Ashworth scale is assessed while moving from extension to flexion.
- *Results Interpretation:* Scores range from 0-4: 0 - No increase in tone 1 - Slight increase in tone giving a catch when the limb was moved in flexion or extension 2 - More marked increase in tone but limb easily flexed 3 - Considerable increase in tone - passive movement difficult 4 - Limb rigid in flexion or extension.

8) Montreal Cognitive Assessment (MoCA) [14]

- *Aim:* To screen cognitive abilities and to detect mild cognitive dysfunction.
- *Test Performance:* The test is a one-page, 30-point test that can be administered in 10 minutes, assessing short-term memory recall, executive functions, concentration, attention, working memory, and language.
- *Results Interpretation:* 18-25 = mild cognitive impairment, – 10-17= moderate cognitive impairment and – less than 10= severe cognitive impairment.

9) EMPATICA wristband [15]

- *Aim:* Measure neurophysiological correlates (Heart Rate, Skin Conductance, Hear Rate Variability, Temperature) of the intervention
- *Test Performance:* The subject will wear the Empatica bracelet while performing the rehabilitation exercises.
- *Results Interpretation:* Demonstrate that the provided intervention is changing and improving neurophysiological parameters (e.g. Decreased Heart Rate).

10) Visual Analogue Scale (VAS) for pain [16]

- *Aim:* To quantify the experience of pain.
- *Test Performance:* The subject marks on a line how much pain they feel.
- *Results Interpretation:* The distance of the mark on the line is measured and compared to the total length of the line to get a fraction of pain. A change of 30% of the length constitutes a clinically significant difference [17].

11) Treatment Satisfaction Measure

- *Aim:* To quantify the satisfaction of patients with the treatment received
- *Test Performance:* The subject's marks on the treatment satisfaction on a 4-point Likert-scale Chart.
- *Results Interpretation:* 1: Very Dissatisfied, 2: Dissatisfied, 3: Satisfied, 4:Very Satisfied

12) Kinematic variables of movement during structured VR tasks

- *Aim:* To assess changes throughout rehabilitation days in kinematic measurements while participants are performing VR tasks
- *Test Performance:* The indices are extracted from the movement data collected during VR tasks
- *Results Interpretation:* The changes in kinematic indexes throughout rehabilitation sessions will be evaluated to have a proxy of improved or deteriorating performances.

2.2 Secondary objective

The secondary objective is to determine whether changes in outcome variables (mobility, sensory assessment, body schema, spatial representation, and functional measures) differ between participants undergoing the VR+TENS intervention and those receiving conventional rehabilitation.

The same outcome measures used for the primary objective will be analyzed, but this time compared between the two intervention groups.

3 Study Description

3.1 Study design

The study has a controlled, parallel design, with two different groups.

3.2 Study centre

The study centre is located at the Clinic for Rehabilitation "Dr. Miroslav Zotović", 11000, Belgrade, Serbia

3.3 Responsible ethics committee

The ethics committee responsible for this study is:

Ethics Committee of the Clinic for Rehabilitation "Dr Miroslav Zotović"

Clinic for Rehabilitation "Dr. Miroslav Zotović"

Sokobanjska 13, Beograd 11000, Serbia

3.4 Registration of the study

After receiving a positive vote from the Ethics Committees of the Clinic for Rehabilitation "Dr Miroslav Zotović, the study will be prospectively registered on clinicaltrial.gov, or a comparable WHO-recognised registry.

3.5 Time schedule

Study-related

Planned start date: 20.04.2024

Planned First Patient Visit: 20.04.2024

Last Possible Patient Visit: 01.06.2026

Patient-related:

Planned active intervention duration: 5-6 weeks. After inclusion, the duration of each patient's participation will be 5-6 weeks, if they can be recruited into the study on the first day (baseline, session to learn about the system, intervention, post-intervention testing).

Please find here an example of the testing, therapy and grand total time per patient

- Total Testing Time (3 Sessions): 510 minutes
- Total Therapy Time (12 Sessions): 720 minutes
- Grand Total per Patient: 1,230 minutes

4 Methods

4.1 Study Population

The contacts with potential participants will be managed by the Clinic for Rehabilitation "Dr. Miroslav Zotović" who will distribute informational materials. Clinic personnel will oversee the formal recruitment process, ensuring that inclusion and exclusion criteria are met. Their expertise will guarantee that participants provide voluntary and informed consent, in full compliance with good clinical practice. We emphasize understanding the trial's purpose, procedures, as well as its potential risks and benefits.

4.1.1 Inclusion and exclusion criteria

Inclusion criteria:

- Stroke patients (ischemic or hemorrhagic)
- At least 3 months after stroke
- Fugl-Meyer Upper Extremity score (FMUE) between 16 and 60.
- The subject should be able to perform a circular movement at the shoulder joint in the frontal plane with the paretic arm which is semiflexed at the elbow (ability to make a circular or semicircular movement with the hand in elbow semiflexion)
- Age between 18 and 80 years.

Exclusion criteria:

- Significant prior neurological and/or psychiatric disorders
- Significant cognitive impairment (MoCA score <10)
- Epilepsy
- Motion sickness
- Pacemakers and/or other electronic implants
- Inability to provide informed consent

4.1.2 Engagement of vulnerable groups

The clinical trial will not be conducted on the following vulnerable groups of patients:

- Pregnant and lactating women;
- Persons who find themselves in an emergency situation, i.e. a state of emergency or who need urgent medical assistance;
- Persons placed in social welfare institutions;
- Persons on mandatory military service;
- Persons deprived of liberty;
- Persons who cannot participate in a clinical trial by court decision;
- Persons who can be influenced to give informed consent by coercion or other means of action.

4.1.3 Number of participants and sample calculation

To determine a priori the sample size necessary to achieve statistical power, we used G*power (http://www.gpower.hhu.de/). The analyses were calculated to achieve a statistical power of

0.90, meaning that we could have a 90% chance of rejecting the null hypothesis. Also, the alpha level for the tests was set at .05, and a two-tailed approach was used. We used the effect size seen for the improvement in FMUE for immersive VR rehabilitation compared to conventional rehabilitation in [18]. This effect size was 1.38.

The t-test with the abovementioned parameters estimated the needed total sample size of 24 (12 for each group) to see a significant improvement of FMUE in the intervention group compared to the conventional rehabilitation. However, considering that there might be drop-outs, and to be as conservative as possible, we increased the number to 40.

4.2 Intervention

4.2.1 Therapy scheme

PERIODS	Name	SCREENING	TREATMENT			FOLLOW-UP
	Duration		3 WEEKS			2 WEEKS
	Name	Screening	Randomization & T0	T1	T2	T3 - Follow-up
	Time	Days/Weeks	Day 0	Week 1 (± 10 days)	Week 3 (± 21 days)	Week 5 (+ 35 days)
**Informed Consent**		X				
**Inclusion / Exclusion Criteria**		X				
**Medical History**		X				
**FMUE**			X	X	X	X
**ARAT**			X	X	X	X
**Barthel Index**			X	X	X	X
**2-point discrimination test**			X	X	X	X
**Body Landmark Test**			X	X	X	X
**Peri-personal Space**			X	X	X	X
**VAS pain**			X	X	X	X
**MOCA test**			X	X	X	X
**Ashworth test**			X	X	X	X
**Satisfaction**					X	

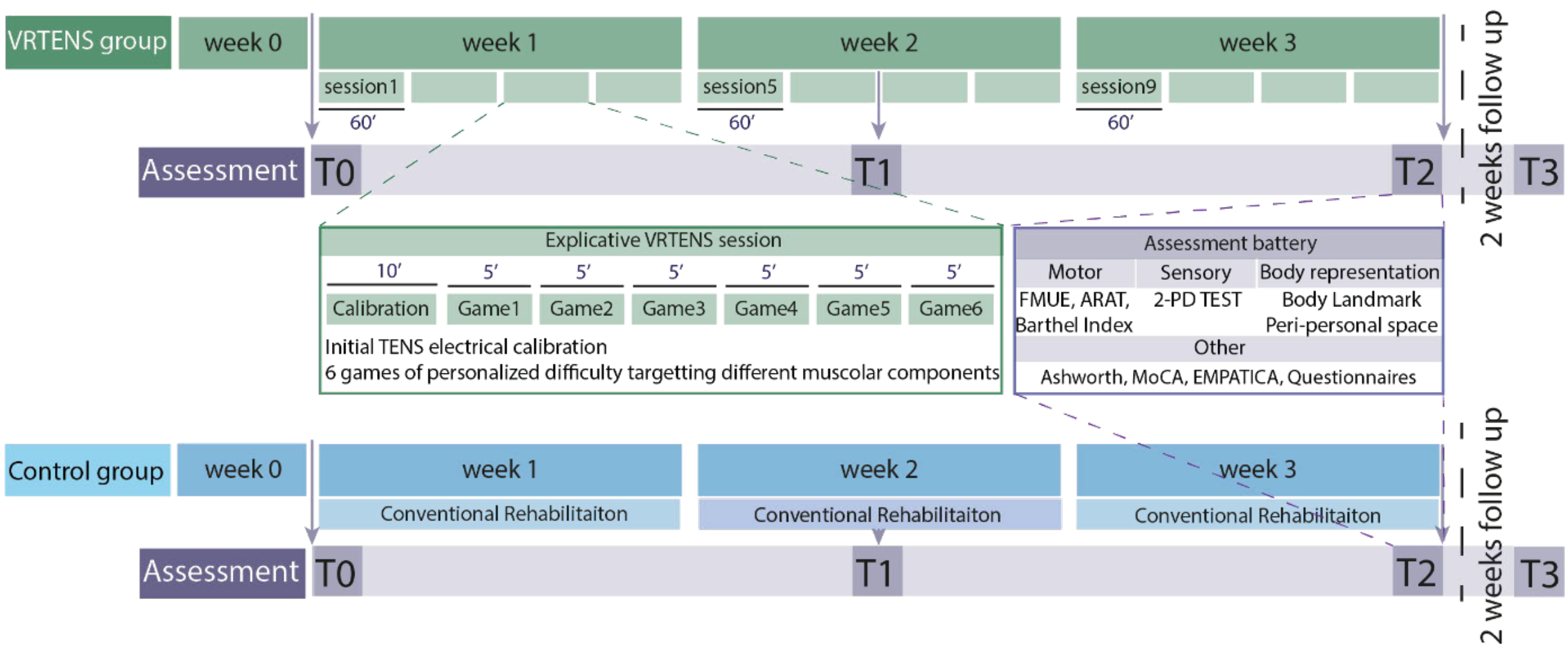

Figure 3: Study Protocol

VR-TENS group

Week 1 – Week 3: During the intervention phase, patients will undergo four 60’ sessions per week of VRTENS rehabilitation. The total number of sessions will be 12. During each of these sessions, the first 10’ will be employed for the calibration of the Transcutaneous Electrical Nerve Stimulation (TENS). In the remaining part of the session, subjects will perform VR-based task-oriented games of personalized difficulty targeting different muscular components. Subjects will perform six games, chosen from all the games offered on the platform. The games will be chosen with clinicians and will depend on the muscular components to rehabilitate.

Here it follows the explanation of each game of the platform. A short description, with the aim and the performance metric of each game, is presented.

1) Shapes Game: Patients will catch specific balls on a predefined shape that will appear in front of them.
   - Aim: Rehabilitate all the shoulder degrees of movement
   - Performance metric: Number of reaching completed, velocity of the movement, smoothness of the movement, and error of the movement.
2) Reaching Game: Patients will reach specific balls that will appear in front of them.
   - Aim: Rehabilitate shoulder flexion/extension.
   - Performance metric: Number of reaching completed, velocity of the movement, smoothness of the movement, and error of the movement.
3) Goalkeeper Game: Patients will be in a soccer field and will save shoots from different angles.
   - Aim: Rehabilitate shoulder adduction/abduction.
   - Performance metric: Number of saves completed, velocity of the movement, smoothness of the movement, and error of the movement.
4) Baseball Game: Patients will grasp a baseball bat to catch specific balls in the air.
   - Aim: Rehabilitate shoulder internal/external rotation.
   - Performance metric: Number of balls touched, velocity of the movement, smoothness of the movement, and error of the movement.
5) Gardening Game: Patients will grasp a watering can and prono-supinate their forearm to garden plants.
   - Aim: Rehabilitate forearm prono/supination.
   - Performance metric: Number of plants gardened, prono-supination ROM, angular velocity of the movement.
6) Biceps Game: Patients will have to catch balls following a biceps curl exercise.

- Aim: Rehabilitate elbow flexion/extension.
- Performance metric: Number of balls touched, velocity of the movement, smoothness of the movement, and error of the movement.

7) Motorbike Game: Patients will have to drive a motorbike to avoid obstacles.
    - Aim: Rehabilitate wrist palmar flexion/extension.
    - Performance metric: Number of tasks completed, palmar flexion/extension ROM, angular velocity of the movement.
8) Cleaning Game: Patients will have to clean a blurred glass.
    - Aim: Rehabilitate wrist radial/ulnar deviation.
    - Performance metric: Number of tasks completed, radial/ulnar deviation ROM, angular velocity of the movement.
9) Grasp Game: Grasp object and release in specific areas.
    - Aim: Rehabilitate opening/closure of the hand.
    - Performance metric: Number of tasks completed, velocity of the movement, smoothness of the movement, and error of the movement.

Control group

Week 1 – Week 3: During the intervention phase, patients will undergo conventional rehabilitation in the clinic, consisting of occupational and physical therapy. The dosage of rehabilitation and types of exercises will be matched with the ones of the VR-TENS group.

Assessment

Both for the VR-TENS group and the Control group, the assessment will be performed at four different time points:

T0: Before the beginning of the intervention, the first day of week 1

T1: At half of the study protocol, after 6 rehabilitation sessions. This would correspond to the half of the second week.

T2: At the end of the intervention. On the last day of week 3

T3: Two weeks after the end of the intervention.

The assessment battery would be the following, already previously described:

1) FMUE [9]
2) ARAT [10]
3) Body Landmark test [3]
4) Barthel Index [11]

5) Two-point discrimination test [12]
6) Peri Personal Space test [3]
7) Ashworth Scale for shoulder, elbow and wrist [13]
8) Montreal Cognitive Assessment (MoCA) [14]
9) Visual Analogue Scale (VAS) for pain [16]
10) Treatment Satisfaction Measure

4.2.2 Assessment of safety and side effects

Subjective and objective safety parameters are collected to assess patient safety. Subjective parameters are those reported by the patient or asked by the therapist. Examples include general sense of well-being, signs of stress, etc.
Constant consultation with the patient regarding these parameters is a standard procedure in the therapy of stroke patients and serves to individually adjust therapy intensity. Objective parameters for assessing safety are those serious or non-serious adverse events that are recorded and evaluated during the study.

4.2.3 Compatibility/ safety parameters

- Subjectively by the patient:

Through close consultation and feedback with the treating physician and therapist (adapting therapy as the situation requires or is possible for the patient – customary in every daily therapy routine).

- Objective parameters:

The recording and evaluation of side effects serves to objectify safety. Continuous monitoring and assessment of the safety of the intervention is carried out to record side effects and adverse events.

4.3 Adverse events

4.3.1 Definitions

The performance of the tests and intervention may trigger adverse events and side effects. However, during therapy, events such as pain, increase in tone, shortness of breath or similar signs of stress may occur as a physiological response to stress.

4.3.2 Definition of adverse events

An adverse event (AE) is any untoward adverse change from the subject's baseline condition, i.e. any unfavourable and unintended sign including an abnormal laboratory finding, symptom or disease which is clinically relevant by the physician that occurs during the course of the study, whether or not considered related to the study intervention.
Adverse events include:

- Exacerbation of a pre-existing disease

- Increase in frequency or intensity of a pre-existing episodic disease or medical condition
- Disease or medical condition detected or diagnosed after the start of the study, even though it may have been present prior to the start of the study
- Continuous persistent disease or symptoms present at baseline that worsen following the start of the study.
- Events considered by the Investigator to be related to study-mandated procedures.
- Abnormal assessments and physical examination findings must be reported as AEs if they represent a clinically significant finding that was not present at baseline or worsened during the course of the study.
- Laboratory test abnormalities must be reported as AEs if they represent a clinically significant finding, symptomatic or not, which was not present at baseline or worsened during the course of the study or led to dose reduction, interruption or permanent discontinuation of study intervention.

Adverse events do not include:

- Medical or surgical procedure, e.g., surgery, endoscopy, tooth extraction, transfusion. However, the event leading to the procedure is an AE. If this event is serious, the procedure must be described in the SAE narrative.
- Pre-existing disease or medical condition that does not worsen.
- Situations in which an adverse change did not occur, e.g., hospitalizations for cosmetic elective surgery or for social and/or convenience reasons.

4.4 Serious adverse events (SAEs)

A Serious Adverse Event (SAE) is defined by the International Conference on Harmonization (ICH) guidelines and GCP guidelines as any AE fulfilling at least one of the following criteria:

- Leads to a death,
- Leads to a serious deterioration in the health of the subject, that resulted in any of the following:
    - life-threatening illness or injury,
    - permanent impairment of a body structure or a body function,
    - hospitalisation or prolongation of patient hospitalisation,
    - medical or surgical intervention to prevent life-threatening illness or injury or permanent impairment to a body structure or a body function,
    - chronic disease
- foetal distress, foetal death or a congenital physical or mental impairment or birth defect.

4.4.1 SAEs related to the intervention

Such SAEs are defined as SAEs that appear to have a reasonable possibility of causal relationship.

4.4.2 Suspected unexpected serious adverse reactions (SUSARs)

SUSARs are all serious adverse reactions with suspected causal relationship to the study intervention that is unexpected (not previously described in the Summary of Product Characteristics or Investigator's brochure) and serious.

4.5 Severity of adverse events

The severity of clinical AEs is graded on a three-point scale: mild, moderate, severe, and reported on specific AE pages of the CRF.

If the severity of an AE worsens during the intervention, only the worst intensity should be reported on the AE page. If the AE lessens in intensity, no change in the severity is required.

Mild

Event may be noticeable to subject; does not influence daily activities; the AE resolves spontaneously or may require minimal therapeutic intervention.

Moderate

Event may make subject uncomfortable; performance of daily activities may be influenced; intervention may be needed; the AE produces no sequelae.

Severe

Event may cause noticeable discomfort; usually interferes with daily activities; subject may not be able to continue in the study; the AE/ADE produces sequelae, which require prolonged therapeutic intervention.

A mild, moderate or severe AE/ADE may or may not be serious. These terms are used to describe the intensity of a specific event (as in mild, moderate, or severe myocardial infarction). However, a severe event may be of relatively minor medical significance (such as severe headache) and is not necessarily serious. For example, nausea lasting several hours may be rated as severe, but may not be clinically serious. Fever of 39°C that is not considered severe may become serious if it prolongs hospital discharge by a day. Seriousness rather than severity serves as a guide for defining regulatory reporting obligations.

4.6 Association with the study intervention

The Investigator will assess the causal relationship between the study intervention and the AE using his/her clinical expertise and judgment according to the following algorithm that best fits the circumstances of the AE:

Unrelated

- May or may not follow a temporal sequence from administration of the study intervention.
- Is biologically implausible and does not follow known response pattern to the suspect the study intervention (if response pattern is previously known).
- Can be explained by the known characteristics of the subject's clinical state or other modes of therapy administered to the subject.

Unlikely

- There is a reasonable temporal relation between the AE and the intake of the study intervention, but there is a plausible other explanation for the occurrence of the AE.

Possibly

- The AE has a reasonable temporal relationship with the study intervention.

- The AE may equally be explained by the study subject's clinically state, environmental or toxic factors, or concomitant therapy administered to the study subject.
- The relationship between study intervention and AE may also be clinically plausible.

Probably

- Follows a reasonable temporal sequence between the AE and the study intervention, and plausible reasons point to a causal relation with the study intervention.

Related

- Follows a reasonable temporal sequence from of the intervention.
- Follows a known response pattern to the intervention (if response pattern is previously known).
- No other reasonable cause is present.

Not assessable

- The causal relationship between the study intervention and the AE cannot be judged.

4.7 Reporting procedures

A special section is designated to adverse events in the case report form. The following details must thereby be entered:

- Type of adverse event
- Start (date and time)
- End (date and time)
- Severity (mild, moderate, severe)
- Serious (no / yes)
- Unexpected (no / yes)
- Outcome (resolved, resolving, not resolved, resolved with sequelae, unknown, fatal)
- Relation to study intervention (Related/ Probably/ Possibly/ Unlikely/ Not related/ Not assessable)

Adverse events are to be documented in the case report form in accordance with the above mentioned criteria.

4.7.1 Reporting procedures for SAEs

In case of a serious adverse event, the Investigator has to use all supportive measures for best patient treatment. A written report is also to be prepared and should at least contain the following:

- Patient number (study code/screening number)
- Patient: age, sex
- The suspected intervention
- The adverse event assessed as serious
- Short description of the event and outcome and any interventions

If applicable, the initial report should be followed by the Follow up report, indicating the outcome of the SAE.

4.7.2 Reporting procedures for SUSAR

It must be remembered that the regulatory authorities, and the Institutional Review Board / Independent Ethics Committee (IRB / IEC) must be informed about all SUSAR. Such reports shall be made by the sponsor and should content at least the following details:

- Patient number (study code/screening number)
- Patient: age in years, sex
- Name of Investigator and investigating site
- Period of administration
- The suspected intervention
- The adverse event assessed as serious and unexpected, and for which there is a suspected causal relationship to the intervention
- Concomitant disease and medication
- Short description of the event:
    - Description
    - Onset and if applicable, end
    - Therapeutic intervention
    - Causal relationship
    - Seriousness criteria or reportable reason

Electronic reporting should be the expected method for reporting of SUSARs to the competent authority. In that case, the format and content as defined by the regulatory requirements should be adhered to. The latest version of MedDRA should be applied. Lower level terms (LLT) should be used.

5 Statistical methodology

5.1 Source data

Source data in this study are informed consent forms, patients' medical records (information about participants' health status regarding stroke), physiological signals, and clinical test results. Project data recorded in are the anonymized participant ID, age, date of participation, inclusion and exclusion criteria, and notes on behavior or technical aspects during the assessments.

5.2 Case Report Form (CRF)

All patient data and examination results will be entered in the custom electronic CRFs (Case Report Forms) specially prepared for this study.
The survey forms may only be filled in with a biro or fineliner (black or blue). Corrections must be made in such a way that the old entry remains legible (the use of correcting agents is not permitted). Corrections must be signed and dated by the authorized person making them. Data that is not available or that has not been collected must be clearly identifiable as such (F or ND). The reasons for this should be documented, if applicable.

The investigator shall ensure that all patient data are entered into the CRFs promptly, legibly, completely, accurately and in accordance with the patient records.

5.3 Data Analysis

5.3.1 Statistical Data Analysis

The statistical analysis will be conducted using MATLAB, and/or Python software. Rehabilitation outcomes, both objective and subjective, will be evaluated across the different tested conditions through non-paired comparisons. Here, it follows the general guidelines applicable to all the statistical tests performed.

1) Normality Test. To assess the distribution of the data, the Kolmogorov-Smirnov test will be applied. This will help determine whether parametric or non-parametric statistical methods are appropriate for further analysis.
2) Comparative Analysis Between the two Groups. Based on the results of the normality test:
    a. If data is normally distributed, an independent t-test will be used to compare outcomes across the two groups.
    b. If data is not normally distributed, the Mann-Whitney test (non-parametric) will be applied for group comparisons.
3) Comparative Analysis Within Groups (across assessment points). Based on the results of the normality test:
    a. If data is normally distributed, repeated measures ANOVA design will be employed, with Least Significant Difference (LSD) post-hoc tests for multiple comparisons.
    b. If data is not normally distributed, a Friedman test design will be employed, with Least Significant Difference (LSD) post-hoc tests for multiple comparisons.
4) Significance Level. Statistical significance will be set at an alpha level of 0.05. All tests will be two-tailed unless otherwise specified.

The following section details the specific tests that will be employed for each outcome of interest. The primary outcomes of the study are improvements in the Fugl-Meyer Upper Extremity (FMUE) scale, Action Research Arm Test (ARAT), and the Body Landmark Task.

FMUE:
For within-group analysis, changes in FMUE scores will not be assessed for statistical significance but will instead be evaluated based on clinical significance, using the minimally clinically important difference (MCID) threshold. Specifically, an improvement of at least 5.25 [19] points on the FMUE will be considered clinically meaningful. This threshold will be applied to evaluate changes from T0 to T1, T2, and T3.

For between-group comparisons, the increase in FMUE scores from T0 to T1, T2, and T3 will be compared between VR-TENS vs Conventional intervention at each assessment point. Depending on the distribution of the data, either an independent t-test (for normally distributed data) or a Mann–Whitney U test (for non-normal data) will be used.

ARAT:
For within-group analysis, changes in ARAT scores will not be assessed for statistical significance but will instead be evaluated based on clinical significance, using the minimally clinically important difference (MCID) threshold. Specifically, an improvement of at least 5.7 [20,21] points on the ARAT will be considered clinically meaningful. This threshold will be applied to evaluate changes from T0 to T1, T2, and T3.
For between-group comparisons, the increase in ARAT scores from T0 to T1, T2, and T3 will be compared between VR-TENS vs Conventional intervention at each assessment point. Depending on the distribution of the data, either an independent t-test (for normally distributed data) or a Mann–Whitney U test (for non-normal data) will be used.

Body Landmark analysis:
For the Body Landmark Test, a statistical within-group analysis will be performed to evaluate changes in body representation. This test involves calculating indices corresponding to the perceived versus actual ratios of arm length and hand width. For each group, data collected across the four assessment points (T0, T1, T2, T3) will be analyzed using a repeated measures ANOVA for normally distributed data, or a Friedman test if normality is not met. Least Significant Difference (LSD) post-hoc tests will be applied to explore pairwise comparisons between time points.

Here it follows a detailed analysis for the other outcomes of the study.

BARTHEL INDEX , ASHWORTH, MOCA
For the Barthel Index, ASHWORT and MOCA scale the same analysis described for the ARAT and FMUE will be used.

2PD analysis:
In this test, participants are touched on the palm of the impaired hand 20 times with either one or two pins at a fixed distance. They are then asked to identify the number of pins. The percentage of correct responses will be calculated as the outcome measure, and the improvement in this success rate will be tested using the following between-group analysis: the increase in 2PD percentage scores from T0 to T1, T2, and T3 will be compared between VR-TENS vs Conventional intervention at each assessment point. Depending on the distribution of the data, either an independent t-test (for normally distributed data) or a Mann–Whitney U test (for non-normal data) will be used.

Peripersonal space analysis:
To analyze changes in PPS across groups, a two-factor within-subjects ANOVA will be performed, with 5 distances × 4 assessment timepoints, to identify significant interaction effects of the reaction times indicative of multisensory facilitation. When a significant

interaction is found, PPS will be calculated at each assessment point as the distance at which reaction times become significantly faster than baseline, using a paired t-test or Wilcoxon

Pain Analysis

For pain evaluation, a more clinically focused approach will be adopted, as not all stroke patients experience pain. Pain levels will be assessed at the beginning and end of the trial for each participant. Clinical significance will be defined as a reduction of at least 2 points on the Visual Analog Scale (VAS) or a 30% decrease in pain. The analysis will report the number of participants who achieved this clinically meaningful reduction rather than group-level statistical comparisons.

EMPATICA wristband:

Mean pulse rate will be measured during each rehabilitation session using the EMPATICA wristband. A within-group analysis will be employed. Pulse rate data collected across rehabilitation days will be analyzed using a repeated measures ANOVA for normally distributed data or a Friedman test for non-normal data. Least Significant Difference (LSD) post-hoc tests will be applied to assess differences between specific days.

Treatment Satisfaction Questionnaire analysis:

For the Treatment Satisfaction Questionnaire, which is rated on a scale from 0 to 10, the analysis will differ slightly. Since this measure is collected only once at the end of the rehabilitation intervention, no improvement will be analyzed. Instead, the single value obtained for each participant will be compared between the two groups using either an independent t-test or the Mann-Whitney U test, depending on the data's normality.

Kinematic Analysis

For all kinematic indicators extracted from movement data (e.g., velocity, acceleration, jerk, smoothness, and tasks per minute), a within-group approach will be employed. Kinematic indices recorded throughout the rehabilitation days will be analyzed using a repeated measures ANOVA for normally distributed data or a Friedman test for non-normal data. Least Significant Difference (LSD) post-hoc tests will be used to assess differences between specific days.

5.4 Storage of study documents, data storage & deletion

The documents required for the clinical trial shall be kept in the study folder. The sponsor and investigator shall take appropriate measures to ensure the careful and confidential handling of all data generated during the study.

Records and documents related to the study (e.g. consent forms, data collection forms, and other relevant documents) shall be retained by the investigators in the original and digital form for at least 5 years after the end or termination of the clinical trial. The investigator as well as the coordinating investigator shall ensure that the documents concerning

pseudonymization are retained for a period of 5 years after the end or termination of the clinical trial. The trial-related data, records and documents shall be stored in secure premises and on secure servers. The medical records and other original data shall be retained for the longest possible period allowed by the institution. Access to the documents must be restricted to the study team members.

5.5 Data Management

5.5.1 Data input and export

The data from questionnaires and survey protocols are compiled and entered by participants. Only data that are specified in the protocol and that are required for the interpretation of the study results are collected in the CRF. The data are then combined in a table format from automatic codes, from which they can be imported into the statistics program.

5.5.2 Quality Ensuring Measures

The following measures are carried out to ensure the plausibility and integrity of the data:
- Documentation of protocol deviations and their evaluation as to whether there is an influence on the data quality.
- Data checking by the PI - prior to data analysis regarding the primary endpoint and secondary endpoints, a recheck for plausibility takes place.

The review of the data focuses on the data on target criteria, patient safety, and protocol deviations.

6 ETHICAL AND LEGAL ASPECTS

6.1 Informed consent of subjects

Following comprehensive instruction regarding the nature, significance, impact and risks of this clinical trial, the patient must give written consent to participation in the study.

During the instruction, the patients are to be made aware of the fact that they can withdraw their consent, without giving reasons, at any time, without their further medical care being influenced in any way.

In addition to the comprehensive instructions given to the patients by the investigator, the patients also receive a written patient information sheet in comprehensible language, explaining the nature and purpose of the study and its progress.

The patients must agree to the possibility of study-related data being passed on to relevant authorities.

The patients must be informed in detail of their obligations in relation to the subject insurance in order not to jeopardize insurance cover.

6.2 Acknowledgement / approval of the study

The Investigator will submit this protocol and any related document provided to the subject (such as subject information used to obtain informed consent) to an Ethics Committee (EC) Approval from the committee must be obtained before starting the study.

6.2.1 Changes in the conduct of the study

Proposed amendments must be submitted to the appropriate CA and ECs. Substantial amendments may be implemented only after CA/EC approval has been obtained. Amendments that are intended to eliminate an apparent immediate hazard to subjects may be implemented prior to receiving CA/EC approval. However, in this case, approval must be obtained as soon as possible after implementation.

6.3 Ethics and good clinical practice (GCP)

The Investigator will ensure that this study is conducted in full conformance with the principles of the "Declaration of Helsinki" (as amended at the 64th WMA General Assembly, Fortaleza, Brazil, 2013) and with the laws and regulations of the country in which the clinical research is conducted.

The Investigator of the clinical trial shall guarantee that only appropriately trained personnel will be involved in the study. All studies must follow the ICH GCP Guidelines and the regulatory requirements.

7 REFERENCES

1. WHO EMRO | Stroke, Cerebrovascular accident | Health topics. World Health Organization - Regional Office for the Eastern Mediterranean. Accessed May 2, 2023. http://www.emro.who.int/health-topics/stroke-cerebrovascular-accident/index.html

2. Edwards LL, King EM, Buetefisch CM, Borich MR. Putting the "Sensory" Into Sensorimotor Control: The Role of Sensorimotor Integration in Goal-Directed Hand Movements After Stroke. *Frontiers in Integrative Neuroscience*. 2019;13. doi:10.3389/FNINT.2019.00016

3. Bassolino M, Franza M, Guanziroli E, et al. Body and peripersonal space representations in chronic stroke patients with upper limb motor deficits. *Brain Commun*. 2022;4(4):fcac179. doi:10.1093/braincomms/fcac179

4. Doyle S, Bennett S, Fasoli SE, McKenna KT. Interventions for sensory impairment in the upper limb after stroke. *Cochrane Database Syst Rev*. 2010;2010(6):CD006331. doi:10.1002/14651858.CD006331.pub2

5. Ingram LA, Butler AA, Brodie MA, Lord SR, Gandevia SC. Quantifying upper limb motor impairment in chronic stroke: a physiological profiling approach. *J Appl Physiol (1985)*. 2021;131(3):949-965. doi:10.1152/japplphysiol.00078.2021

6. Odermatt IA, Buetler KA, Wenk N, et al. Congruency of Information Rather Than Body Ownership Enhances Motor Performance in Highly Embodied Virtual Reality. *Frontiers in Neuroscience*. 2021;15:711. doi:10.3389/FNINS.2021.678909/BIBTEX

7. Matamala-Gomez M, Slater M, Sanchez-Vives MV. Impact of virtual embodiment and exercises on functional ability and range of motion in orthopedic rehabilitation. *Scientific Reports*. 2022;12(1). doi:10.1038/s41598-022-08917-3

8. Matamala-Gomez M, Malighetti C, Cipresso P, et al. Changing Body Representation Through Full Body Ownership Illusions Might Foster Motor Rehabilitation Outcome in Patients With Stroke. *Frontiers in Psychology*. 2020;11. doi:10.3389/fpsyg.2020.01962

9. Gladstone DJ, Danells CJ, Black SE. The fugl-meyer assessment of motor recovery after stroke: a critical review of its measurement properties. *Neurorehabil Neural Repair*. 2002;16(3):232-240. doi:10.1177/154596802401105171

10. HSIEH CL, HSUEH IP, CHIANG FM, LIN PH. Inter-rater reliability and validity of the Action Research arm test in stroke patients. *Age and Ageing*. 1998;27(2):107-113. doi:10.1093/ageing/27.2.107

11. Barthel Index - an overview | ScienceDirect Topics. Accessed February 21, 2024. https://www.sciencedirect.com/topics/medicine-and-dentistry/barthel-index

12. Two-Point Discrimination - an overview | ScienceDirect Topics. Accessed February 21, 2024. https://www.sciencedirect.com/topics/agricultural-and-biological-sciences/two-point-discrimination

13. Lee KC, Carson L, Kinnin E, Patterson V. The Ashworth Scale: A Reliable and Reproducible Method of Measuring Spasticity. *Journal of Neurologic Rehabilitation*. 1989;3(4):205-209. doi:10.1177/136140968900300406

14. Wong A, Law LSN, Liu W, et al. Montreal Cognitive Assessment: One Cutoff Never Fits All. *Stroke*. 2015;46(12):3547-3550. doi:10.1161/STROKEAHA.115.011226

15. McCarthy C, Pradhan N, Redpath C, Adler A. Validation of the Empatica E4 wristband. In: *2016 IEEE EMBS International Student Conference (ISC)*. ; 2016:1-4. doi:10.1109/EMBSISC.2016.7508621

16. Delgado DA, Lambert BS, Boutris N, et al. Validation of Digital Visual Analog Scale Pain Scoring With a Traditional Paper-based Visual Analog Scale in Adults. *JAAOS Global Research & Reviews*. 2018;2(3):e088. doi:10.5435/JAAOSGlobal-D-17-00088

17. Dworkin RH, Turk DC, Wyrwich KW, et al. Interpreting the Clinical Importance of Treatment Outcomes in Chronic Pain Clinical Trials: IMMPACT Recommendations. *The Journal of Pain*. 2008;9(2):105-121. doi:10.1016/j.jpain.2007.09.005

18. Hao J, He Z, Yu X, Remis A. Comparison of immersive and non-immersive virtual reality for upper extremity functional recovery in patients with stroke: a systematic review and network meta-analysis. *Neurol Sci*. 2023;44(8):2679-2697. doi:10.1007/s10072-023-06742-8

19. Page SJ, Fulk GD, Boyne P. Clinically Important Differences for the Upper-Extremity Fugl-Meyer Scale in People With Minimal to Moderate Impairment Due to Chronic Stroke. *Physical Therapy*. 2012;92(6):791-798. doi:10.2522/ptj.20110009

20. Lang CE, Wagner JM, Dromerick AW, Edwards DF. Measurement of Upper-Extremity Function Early After Stroke: Properties of the Action Research Arm Test. *Archives of Physical Medicine and Rehabilitation*. 2006;87(12):1605-1610. doi:10.1016/j.apmr.2006.09.003

21. van der Lee JH, Beckerman H, Lankhorst GJ, Bouter LM. The responsiveness of the Action Research Arm test and the Fugl-Meyer Assessment scale in chronic stroke patients. *J Rehabil Med*. 2001;33(3):110-113. doi:10.1080/165019701750165916
