# Supplementary material for: Immersive virtual reality with synchronous neurostimulation for upper-limb recovery after stroke: a randomized feasibility trial
Source: Nat Med. 2026 Jun 26;32(8):2790–802. doi: 10.1038/s41591-026-04486-4 (PMC13472961; doi:10.1038/s41591-026-04486-4)
Supplement: Supplementary file 2 [file 41591_2026_4486_MOESM2_ESM.pdf]

nature portfolio

Corresponding author(s): Stanisa Raspopovic

Last updated by author(s): May 20, 2026

Reporting Summary

Nature Portfolio wishes to improve the reproducibility of the work that we publish. This form provides structure for consistency and transparency in reporting. For further information on Nature Portfolio policies, see our Editorial Policies and the Editorial Policy Checklist.

Statistics

For all statistical analyses, confirm that the following items are present in the figure legend, table legend, main text, or Methods section.

n/a	Confirmed	
☐	☒	The exact sample size (*n*) for each experimental group/condition, given as a discrete number and unit of measurement
☐	☒	A statement on whether measurements were taken from distinct samples or whether the same sample was measured repeatedly
☐	☒	The statistical test(s) used AND whether they are one- or two-sided<br>*Only common tests should be described solely by name; describe more complex techniques in the Methods section.*
☒	☐	A description of all covariates tested
☐	☒	A description of any assumptions or corrections, such as tests of normality and adjustment for multiple comparisons
☐	☒	A full description of the statistical parameters including central tendency (e.g. means) or other basic estimates (e.g. regression coefficient) AND variation (e.g. standard deviation) or associated estimates of uncertainty (e.g. confidence intervals)
☐	☒	For null hypothesis testing, the test statistic (e.g. *F*, *t*, *r*) with confidence intervals, effect sizes, degrees of freedom and *P* value noted<br>*Give P values as exact values whenever suitable.*
☒	☐	For Bayesian analysis, information on the choice of priors and Markov chain Monte Carlo settings
☒	☐	For hierarchical and complex designs, identification of the appropriate level for tests and full reporting of outcomes
☐	☒	Estimates of effect sizes (e.g. Cohen's *d*, Pearson's *r*), indicating how they were calculated

Our web collection on statistics for biologists contains articles on many of the points above.

Software and code

Policy information about availability of computer code

Data collection	No software was used for collecting clinical data. VR-related data were collected using Unity (version 2021.3, Unity Technologies) with standard C# scripting approaches.
Data analysis	Data were analyzed using custom scripts written in MATLAB (R2024a, MathWorks) and Python (version 3.13).<br>The custom code used to process and analyse the data and generate the figures is available on Zenodo DOI:10.5281/zenodo.16043228.

For manuscripts utilizing custom algorithms or software that are central to the research but not yet described in published literature, software must be made available to editors and reviewers. We strongly encourage code deposition in a community repository (e.g. GitHub). See the Nature Portfolio guidelines for submitting code & software for further information.

Data

Policy information about availability of data

All manuscripts must include a data availability statement. This statement should provide the following information, where applicable:

- Accession codes, unique identifiers, or web links for publicly available datasets
- A description of any restrictions on data availability
- For clinical datasets or third party data, please ensure that the statement adheres to our policy

The data that support the findings of this study are available on Zenodo DOI:10.5281/zenodo.16043228. The study protocol is included in the Supplementary Information.

Research involving human participants, their data, or biological material

Policy information about studies with human participants or human data. See also policy information about sex, gender (identity/presentation), and sexual orientation and race, ethnicity and racism.

Reporting on sex and gender	Only sex (biological attribute) was recorded based on participant self-report. Gender identity was not collected. Analyses were not disaggregated by sex, as no sex-related effects were hypothesized or observed.
Reporting on race, ethnicity, or other socially relevant groupings	Race, ethnicity, socioeconomic status, and education level were not collected or used in the analyses, as they were not considered relevant to the study objectives. Datasets were not constructed on the basis of race, ethnicity, or other socially relevant groupings.
Population characteristics	Participants were ischemic or haemorrhagic stroke survivors at least 3 months post-stroke, with upper-limb motor impairment (Fugl-Meyer Upper Extremity score between 10 and 60).
Recruitment	Participants were recruited at the Clinic for Rehabilitation “Dr. Miroslav Zotović” in Belgrade, Serbia. Clinic staff identified eligible patients based on clinical records. Potential biases include self-selection (those more motivated or with milder impairments might be more likely to enroll). All eligible patients were approached to minimized selection bias.
Ethics oversight	Ethical Committee of Clinic for Rehabilitation “Dr. Miroslav Zotović”

Note that full information on the approval of the study protocol must also be provided in the manuscript.

Field-specific reporting

Please select the one below that is the best fit for your research. If you are not sure, read the appropriate sections before making your selection.

☒ Life sciences ☐ Behavioural & social sciences ☐ Ecological, evolutionary & environmental sciences

For a reference copy of the document with all sections, see nature.com/documents/nr-reporting-summary-flat.pdf

Life sciences study design

All studies must disclose on these points even when the disclosure is negative.

Sample size	To determine a priori the sample size necessary to achieve statistical power, we used G*power (http://www.gpower.hhu.de/). The analyses were calculated to achieve a statistical power of 0.90, meaning that we could have a 90% chance of rejecting the null hypothesis. Also, the alpha level for the tests was set at 05, and a two-tailed approach was used. We used the effect size seen for the improvement in FMUE for immersive VR rehabilitation compared to conventional rehabilitation in DOI: 10.1007/s10072-023-06742-8 . This effect size was 1.38. The t-test with the abovementioned parameters estimated the needed total sample size of 24 (12 for each group) to see a significant improvement of MUE in the intervention group compared to the conventional rehabilitation. However, considering that there might be dropouts, and to be as conservative as possible, we increased the number to 40.
Data exclusions	No data were excluded from the analysis. All participants who completed baseline and post-intervention assessments were included.
Replication	The VR intervention and assessment procedures were standardized and applied consistently across all participants in both groups. The analyses were based on pre-specified outcomes, and no findings failed internal reproducibility within the trial.
Randomization	Randomization was conducted using a custom MATLAB script provided by an independent third party to ensure allocation concealment.
Blinding	Due to the nature of the study (MultiSensy vs conventional therapy), investigators were not masked to group allocation, as they were involved in the rehabilitation intervention.

Reporting for specific materials, systems and methods

We require information from authors about some types of materials, experimental systems and methods used in many studies. Here, indicate whether each material, system or method listed is relevant to your study. If you are not sure if a list item applies to your research, read the appropriate section before selecting a response.

Materials & experimental systems

n/a	Involved in the study
☒	☐ Antibodies
☒	☐ Eukaryotic cell lines
☒	☐ Palaeontology and archaeology
☒	☐ Animals and other organisms
☐	☒ Clinical data
☒	☐ Dual use research of concern
☒	☐ Plants

Methods

n/a	Involved in the study
☒	☐ ChIP-seq
☒	☐ Flow cytometry
☒	☐ MRI-based neuroimaging

Clinical data

Policy information about clinical studies
All manuscripts should comply with the ICMJE guidelines for publication of clinical research and a completed CONSORT checklist must be included with all submissions.

Clinical trial registration
NCT06400823 (clinicaltrial.gov)

Study protocol
The study protocol is provided as supplementary material.

Data collection
Data collection took place at the Clinic for Rehabilitation "Dr. Miroslav Zotovic" in Belgrade, Serbia. Recruitment and data collection occurred between April 2024 and April 2025. Standardized procedures were followed for all clinical assessments and VR-based kinematic measurements.
Outcomes data were collected at 4 assessment points:

TO: before the intervention starts, on the first day of week 1
T1: at the midpoint of the study protocol, after 6 rehabilitation sessions, week 2
T2: at the end of the intervention
T3: two weeks after the end of the intervention

Outcomes
Primary and secondary outcomes were pre-defined based on the study objectives and specified before data collection, together with the four assessment time points (T0, T1, T2, T3). Primary outcomes were selected to capture the main expected effects on upper-limb motor function and body representation, and included FMUE, ARAT, and the Body Landmark Test. Secondary outcomes assessed complementary domains, including functional independence, tactile acuity, peripersonal space, and objective kinematic performance, using the Barthel Index, Two-Point Discrimination test, Peripersonal Space test, and VR-derived kinematic measures.

Plants

Seed stocks
Report on the source of all seed stocks or other plant material used. If applicable, state the seed stock centre and catalogue number. If plant specimens were collected from the field, describe the collection location, date and sampling procedures.

Novel plant genotypes
Describe the methods by which all novel plant genotypes were produced. This includes those generated by transgenic approaches, gene editing, chemical/radiation-based mutagenesis and hybridization. For transgenic lines, describe the transformation method, the number of independent lines analyzed and the generation upon which experiments were performed. For gene-edited lines, describe the editor used, the endogenous sequence targeted for editing, the targeting guide RNA sequence (if applicable) and how the editor was applied.

Authentication
Describe any authentication procedures for each seed stock used or novel genotype generated. Describe any experiments used to assess the effect of a mutation and, where applicable, how potential secondary effects (e.g. second site T-DNA insertions, mosiacism, off-target gene editing) were examined.
